# Lignin Nanosphere-Supported Cuprous Oxide as an Efficient Catalyst for Huisgen [3+2] Cycloadditions under Relatively Mild Conditions

**DOI:** 10.3390/polym10070724

**Published:** 2018-07-02

**Authors:** Zidan Zhou, Xinwen Peng, Linxin Zhong, Xuehui Li, Runcang Sun

**Affiliations:** 1 State Key Laboratory of Pulp and Paper Engineering, South China University of Technology, Guangzhou 510641, China; zidanzhou@aliyun.com; 2 School of Chemistry and Chemical Engineering, South China University of Technology, Guangzhou 510641, China; cexhli@scut.edu.cn; 3 Beijing Key Laboratory of Lignocellulosic Chemistry, Beijing Forestry University, Beijing 100083, China; rcsun3@bjfu.edu.cn

**Keywords:** electrospraying technology, lignin, nanospheres, solvent-free reaction

## Abstract

In this work, low-cost lignin nanospheres were fabricated and further applied as an efficient and sustainable support for preparing cuprous oxide (Cu_2_O) “green” catalyst by using electrospraying technology. The unalloyed lignin, a special three-dimensional molecular structure, was successfully processed into uniform nanospheres under an electrospraying condition. The synthesized lignin-supported Cu_2_O catalyst had a well-defined nanosphere structure, and Cu_2_O nanoparticles with sizes less than 30 nm were supported by exposed layers of lignin nanospheres. There were C–O–Cu bonds formed between the lignin nanospheres and the metallic nanoparticles. The lignin nanospheres and the lignin nanosphere-supported catalyst werfe characterized by utilizing XRD, SEM, TEM, XPS, EDS, and TGA. The immobilization of Cu_2_O nanoparticles on the lignin nanospheres was beneficial for dispersion of the Cu_2_O nanoparticles and preventing their aggregation, which could cause catalyst deactivation, which favored the Huisgen [3+2] cycloaddition reaction. The triazole synthesis results indicated that the lignin nanosphere-supported Cu_2_O catalyst had a high catalytic performance with 99% yield under solvent-free conditions. Furthermore, the as-synthesized catalyst could be recycled for four times without significantly losing its catalytic activity.

## 1. Introduction

“Click” chemistry has a wide scope, gives high yields, and forms irreversible carbon–heteroatom and carbon–carbon bonds [1]. It uses only the most practical and reliable chemical reactions to connect a diversity of structures bearing a wide variety of functional groups [2]. Therefore, it has attracted much attention in many research areas, such as catalyst design, polymer synthetization, material science, synthesizing libraries of compounds, and drug development [3]. Monovalent copper-mediated azide–alkyne Huisgen [3+2] cycloaddition is a highly typical and important “click” reaction [4]. It involves the ligation of azides and terminal alkynes to generate triazoles, and usually needs a copper salt in conjunction with a base. The catalyst can be a Cu(I) salt or Cu(I) generated in situ by the reduction of Cu(II) salts, usually in organo-aqueous media [5], and allows facile and reliable production of 1,4-disubstituted 1,2,3-triazoles.

Nowadays, there is a growing interest in the synthesis of environmentally benign catalysts [6] because of their added advantages, such as requiring mild reaction conditions and being simple to recover and regenerate, as well as being environmentally friendly [7]. It is desirable to develop high-performance and “green” heterogeneous catalysts for “click” chemistry. Recently, the development of Cu-based heterogeneous catalysts with the presence of copper or cuprous oxide nanoparticles has significantly increased catalytic activity for the construction of triazole molecules, owing to the enhanced surface area of copper or cuprous oxide and unique catalyst structure–activity relationships [8,9,10]. The supported catalysts are simple to prepare. The inexpensive catalysts are practical for their applications. Moreover, the immobilization of small Cu or Cu_2_O nanoparticles on supports makes the catalyst easily reusable with excellent recyclability performance, largely reduces nanoparticle leakage, and avoids environmental hazards. Currently, the most commonly used solid supports for heterogeneous catalysts include silica [11], zeolites [12], graphene [13], magnetic materials [14], and soluble and insoluble polymers [15]. Furthermore, various other supports have been used in different catalytic reactions [16,17,18,19,20,21]. However, some of these supports of the heterogeneous catalysts may not be sufficient in the near future. As a result, it is crucial to switch to cheap and biorenewable resources. Natural biopolymers [22,23,24] have been considered as attractive candidates to create high-performance biobased catalysts due to their abundance, renewability, biodegradability, and low cost [25]. Lignin is one of the most important biopolymers from renewable resources, owing to its abundance (being the second most plentiful plant biopolymer on the earth, after cellulose). The production of lignin is over 50 million tons worldwide annually, while its utilization is rather limited except for its use in biochar or activated carbon [26]. Therefore, lignin is a desirable raw material for fabricating value-added products for various applications. Recently, the synthesis of lignin-based functional materials and their potential applications have attracted much attention, such as anode materials for lithium-ion batteries [27], supercapacitors [28], and electrocatalysts [29]. Furthermore, lignin derivatives exhibit interesting and attractive abilities in improving the performance of photovoltaic devices [30]. As a heterogeneous and rigid polymer of phenolic nature, it is the only biomass that is based on aromatic units. It is composed of phenylpropane, including *p*-coumaryl alcohol, coniferyl alcohol, and sinapyl alcohol, which makes it highly polar with a large amount of hydroxyl groups. Moreover, typical C–O links of the lignin are β–*O*–4, α–*O*–4, and 4–*O*–5; and C–C links are β–5, 5–5, β–1, and β–β linkages [31,32,33,34]. It has hydroxylic groups and aromatic methoxy, carboxylic, carbonylic, and ethereal moieties. Most importantly, lignin owns three-dimensional molecular structures. These make lignin an efficient and stable ligand for the preparation of biocatalysts.

Electrospinning is a relatively convenient and versatile strategy to produce nanofibers from polymer solutions or blends. However, lignin possesses complex three-dimensional structures with various types of functional groups. The lignin is formed by oxidative coupling of the main three kinds of lignin precursors; the reaction sites abound upon forming of the body polymers. The special architectural structures of lignin have led to little success in creating absolute lignin electrospinning nanofibers. Current strategies for using lignin in electrospinning nanofibers focus on integrating it with other natural or synthetic polymers; for instance, Kai et al. [35] engineered nanofibrous composites with different lignin mass fractions into lignin–polymethylmethacrylate by electrospinning technology. Interestingly, we found that the special molecular structure of lignin made it easy to be processed into nanospheres when electrospraying lignin precursor solution at an appropriate concentration in DMF solvent under a determined voltage condition. The inherent phenolic hydroxyl, carboxylic, and carbonylic groups in lignin molecules are beneficial for the adsorption of Cu ions, among others, to load and coordinate [36,37], and the three-dimensional molecular structure contributes to making lignin a stable and favorable support for catalysts.

Herein, we report a novel straightforward method to fabricate lignin nanospheres in quantity (in the grams scale) by utilizing electrospraying technology. Then, we successfully exploit it as an effective support for Cu_2_O nanoparticles for preparing a high-performance cuprous oxide heterogeneous catalyst. Above all, lignin and monovalent copper could form Cu_2_O@L composites owing to the abundant carbon–oxygen bonds in lignin molecules. Meanwhile, the strongly polar phenolic hydroxyl and alcoholic hydroxyl groups contributed to its reducing property [38,39], which favored the reduction of cupric sulfate in aqueous solution for the preparation of cuprous oxide catalyst. In particular, Cu_2_O@L was investigated to catalyze a series of Huisgen “click” reactions and showed excellent activity for 1,2,3-triazole syntheses. The reactions proceeded under air conditions in the absence of organic solvents, without adding any base or external heating, which made the method to be a simple, powerful, and environmentally benign alternative. Furthermore, the catalytic performance of the Cu_2_O@L was researched in terms of a series of azides and terminal alkynes. As compared with other copper-mediated Huisgen “click” cycloaddition reactions, “click” reactions using the Cu_2_O@L exhibited a higher yield and required a shorter reaction time and relatively mild reaction conditions.

## 2. Materials and Methods

The alkali lignin (M_W_ = 6000) was purified from poplar wood pulping black liquor using acid treatment; the black liquor was provided by Shuntai Co., Ltd. (Changsha, Hunan, China). Cu_2_O (~30 nm) for comparison was purchased from Shandong Xiya Chemical Reagent Co., Ltd. (Linyi, Shandong, China). All the chemicals were used as received without further purification.

### 2.1. Electrospraying Lignin Nanopheres

In a typical process, lignin solution with a concentration of 35 wt % was prepared by dissolving 0.9 g lignin into 1.1 g DMF liquid solution in a flask. The mixture liquid was then stirred at room temperature for 2 h until the lignin was completely dissolved. The electrospraying setup used in this study consisted of a syringe and needle (ID = 0.84 mm), high voltage supply, a ground electrode, and a collector (stainless steel sheet). A syringe pump connected to the syringe controlled the flow rate. The lignin nanospheres were obtained by electrospraying at a positive voltage of 15 kV and a negative voltage of 1 kV, with a tip-to-collector distance of 15 cm and a solution flow rate of 0.75 mL/h. The procedure was carried out at 25 °C and 60% ambient humidity. The lignin nanospheres were stripped down from aluminum paper and then dried in an oven. Furthermore, the electrosprayed lignin nanospheres were preliminarily heated at 280 °C in air for 2 h and then were heated to 800 °C with a heating rate of 1 °C/min. Finally, the nanospheres were held for 30 min under N_2_ atmosphere in a tube furnace to obtain lignin carbon nanospheres.

### 2.2. Preparation of Cu_2_O@L

The preparation of Cu_2_O@L used the methods as follows: In a 50 mL test tube, 10 mL of double-distilled water was added into the test tube and stirred at room temperature. 500 mg lignin nanospheres were added to form a suspension. Then, 10 mL (2%, *w*/*v*) of aqueous copper sulphate solution was added into the suspension with stirring for an hour. This process was carried out in N_2_ atmosphere to eliminate the dissolved oxygen present in the solution. An aqueous solution of hydrazine hydrate (4 M) as the main reducing agent was added to reduce the copper sulfate to Cu_2_O nanoparticles sufficiently. After 2 h of continuous stirring, the catalyst was separated using a centrifuge (10,000 rpm, 10 min) and washed with double-distilled water for three times to remove the unentrapped particles and ions. The wet sample was then dried in an oven to obtain Cu_2_O@L.

### 2.3. Catalytic Performance Test

The catalytic ability of Cu_2_O@L was evaluated by the synthesis of triazoles from alkyl azide and alkyne. 1.2 mmol of alkyne, 1.0 mmol of alkyl azide, and 20 mg (1.2 mol %) Cu_2_O@L were added into a reaction tube under organic solvent-free conditions. Then, the reaction mixture was stirred for 3 h at room temperature. After the reaction, the product was separated by ethyl acetate extraction from Cu_2_O@L, and the catalyst was recovered by filtration or centrifugation (8000 rpm, 10 min) for reuse. The products were further purified by column chromatography on silica gel using ether/ethyl acetate (5:1) as the eluent. The triazoles were obtained after solvent evaporation and the yield of the products was calculated.

For the practical applications of such heterogeneous systems, the lifetime of the catalyst is a very significant factor for evaluation of catalyst performance. Therefore, the number of cycles and the catalytic activity in terms of reaction yield using the recycled catalyst were also studied. After the completion of the first reaction, the product was extracted using ethyl acetate and the catalyst was recovered by simple centrifugation and dried at room temperature. Then, a fresh reaction was performed with new reactants, under the same conditions.

### 2.4. Analyses

All ^1^H spectra of the products were recorded on a FT-NMR (600 MHz, Bruker Corp., Karlsruhe, German) spectrometer, and ^13^C spectra were recorded on a Bruker FT-NMR (151 MHz). NMR chemical shifts are given as δ values (ppm) with reference to TMS as the internal standard. FTIR spectra were recorded on a Bruker Nicolet 6700 FTIR spectrophotometer in the range of 400–4000 cm^−1^ using KBr pellets. The Cu content was determined by a Hitachi Z-2000 AAS (Tokyo, Japan). SEM images were recorded on a Zeiss EVO-18 (Oberkochen, Germany) operating at 10 kV, and TEM images were recorded on a JEM-2100 (HR) TEM (JEOL, Tokyo, Japan) working at 200 kV. The fluorescence images were acquired using a LSCM (Leica TCS SP5, Wetzlar, Germany) equipped with a diode laser (405 nm), using a 100× oil objective with numerical aperture of 1.40, and three samples were observed under the same conditions. The UV–vis–NIR absorption spectrum of Cu_2_O was acquired with a PerkinElmer Lambda 750 spectrophotometer (Waltham, MA, USA). FL were taken on a FLS-980 spectrometer (Edinburgh Instruments, Ltd., Edinburgh, UK). The samples were excited by a pulse laser (405 nm). An appropriate filter was also used before the PMT to cut off any other stray light. Topographic and phase images of samples were obtained using an AFM (Bruker Dimension Fastscan) in tapping mode. XRD measurements were carried out at room temperature using a Bruker D8 Advance X-ray powder diffractometer with Ni-filtered Cu-Kα radiation (λ = 0.154 nm) from 5°–80°. XPS measurements were performed on an Axis Ultra DLD instrument using Al-Kα radiation (hν = 1486.6 eV) with contaminated C as an internal standard (C 1s = 284.6 eV). EDS was measured with a Horiba XMAX X-act energy dispersive spectroscope that was attached to the Oxford Instruments (Abingdon, UK). The size and the size distribution of Cu_2_O@L were measured by dynamic light scattering on a Nano-Zetasizer (Zetasizer NanoZS, Malvern Instruments Ltd., Worcestershire, UK) at 25 °C under a scattering angle of 173° at λ = 633 nm. TGA (Q500, TA Instruments, New Castle, DE, USA) was carried out in an aluminum crucible by heating to 650 °C at a heating rate of 20 °C min^−1^ with a nitrogen flow of 25 mL min^−1^.

## 3. Results

### 3.1. Characterization of the Electrosprayed Lignin Nanosphere and Cu_2_O@L

As shown in Figure S1, the concentration of the lignin solution shows a significant influence on the lignin morphology under the electrospraying condition. When the concentration of the lignin solution is 25%, 35%, or 45% (*w*/*w*), electrosprayed lignin exhibits a nanosphere shape (Figure S1a–c). Under these concentration conditions, the surface tension is greater than the electric force, which plays a key role on the nanospherical shape of lignin. However, the electrosprayed lignin exhibits the similar fiber morphology when the lignin concentration is higher than 55%, due to lignin molecular chains twining around each other (Figure S1d). As compared with the electrosprayed lignin at other concentrations, the lignin nanosphere at the concentration of 35% solution had a more uniform morphology and therefore was chosen for further studies.

**Figure 1:**
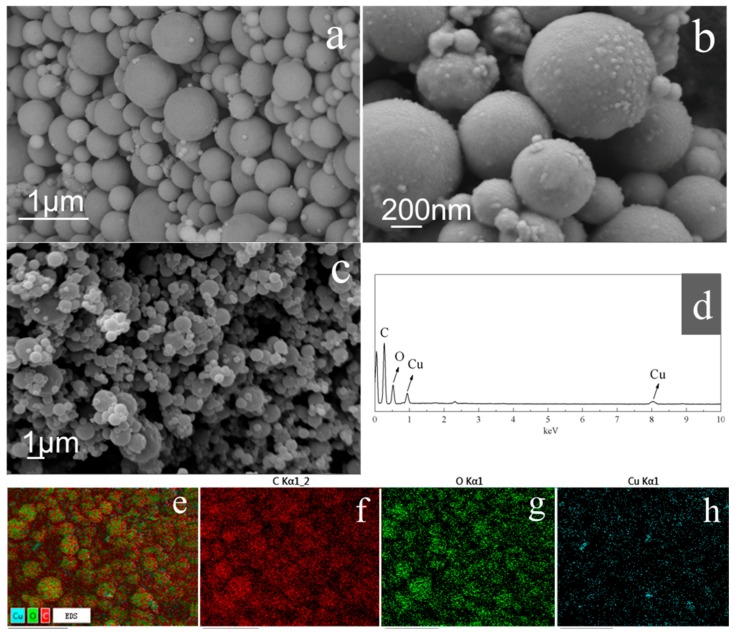
SEM images of lignin nanospheres and Cu_2_O@L: (**a**) electrosprayed lignin nanospheres; (**b**) Cu_2_O@L; (**c**) lignin nanosphere after carbonization at 800 °C under N_2_ atmosphere; (**d**) the EDS of Cu_2_O@L; (**e**) EDS mapping images of C, O, and Cu elements; and (**f**) C element, (**g**) O element, and (**h**) Cu element EDS mapping, respectively.

The as-prepared electrosprayed lignin nanospheres show a regular spherical morphology with a diameter less than 1 μm for the most part (Figure 1a). From the SEM images, the sizes of lignin nanospheres show the average size of 605 ± 5 nm. As shown in Figure 1b, Cu_2_O@L shows a “raspberry” morphology. Furthermore, the spherical morphology of the electrosprayed lignin nanosphere still remains after carbonization at 800 °C under N_2_ atmosphere (Figure 1c). The reason for this is that the good rigid and three-dimensional molecular structure obviously maintains its volume unchanged before and after the carbonization process. The EDS of Cu_2_O@L illustrates that there are C, O, and Cu elements in the catalyst (Figure 1d). Figure 1e shows the EDS mapping image with all the elements of Cu_2_O@L, while Figure 1f–h shows the EDS mapping images of the C, O, and Cu elements, respectively. The SEM and EDS results showed that the Cu_2_O@L was a spherical structure containing the Cu element. Furthermore, the Cu concentration of the catalyst is confirmed to be 7.76% by AAS.

**Figure 2:**
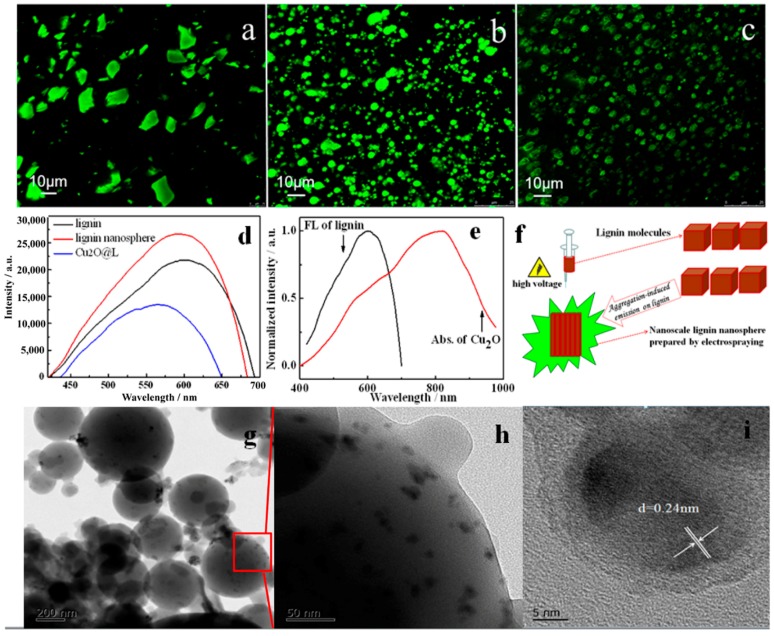
LSCM images, fluorescence spectra, UV–vis–NIR absorption, and TEM images of samples: (**a**) LSCM image of raw lignin; (**b**) LSCM image of lignin nanospheres; (**c**) LSCM image of raw Cu_2_O@L; (**d**) fluorescence spectra of lignin, lignin nanospheres, and Cu_2_O@L; (**e**) UV–vis–NIR absorption of Cu_2_O and lignin; (**f**) proposed aggregation-induced emission of lignin nanospheres prepared by electrospraying; (**g**) TEM image of Cu_2_O@L; (**h**) the expansion of a certain part of image of g, and (**i**) HRTEM image of the nanoparticles on lignin nanospheres.

The morphology of the electrosprayed lignin nanospheres and Cu_2_O@L were further studied using LSCM. As shown in Figure 2a, the raw lignin has an irregular shape and good fluorescence characteristic, which is attributed to its molecular structure containing repeated units of phenylpropane. Interestingly, as compared with the electrosprayed lignin nanospheres (Figure 2b), Cu_2_O@L shows weaker fluorescence (Figure 2c). Fluorescence spectrums of lignin, lignin nanospheres, and Cu_2_O@L were further investigated, which were in accordance with the results of LSCM. The lignin nanospheres show stronger fluorescence intensity than raw lignin. This is probably because the aggregation-induced emission appears among lignin molecules as the lignin is processed into nanospheres by electrospraying. The Cu_2_O nanoparticles can lead to fluorescent quenching of lignin (Figure 2d), and energy transfer from lignin to Cu_2_O nanoparticles occurs owing to some overlap between the emission wavelength range of lignin (420–700 nm) and the absorption wavelength of Cu_2_O (400–900 nm) (Figure 2e). Furthermore, the interaction of phenolic hydroxyl groups and carbon–oxygen and cuprous bonds destroying conjugated structures or clusters of the carbonyl groups in lignin molecules [40] may result in the weakening of the characteristic fluorescence of the lignin and an obvious blue-shift of the luminescent spectrum of Cu_2_O@L. Aggregation-induced emission of lignin nanospheres that were prepared by electrospraying is observed (Figure 2f), similarly to the phenomenon of lignin derivatives reported by Qiu and coworkers [41].

Figure 2 shows TEM and high-resolution TEM (HRTEM) images of Cu_2_O@L as well. The diameter of the electrosprayed lignin nanospheres is less than 1 μm for the most part and some nanoparticles are present on their surfaces, as illustrated in Figure 2g. Noting that the Cu_2_O are irregular in shape with the average size of 20 ± 2 nm (Figure 2h). The HRTEM image (Figure 2i) shows that the lattice spacing of the nanoparticles is about 0.24 nm [42], which is in agreement with the crystal lattice theoretical value for the (111) planes of Cu_2_O. The result indicates that the nanoparticles on lignin containing the Cu element are Cu_2_O. All these results show that small Cu_2_O nanoparticles have been successfully loaded onto the surfaces of the lignin nanospheres.

**Figure 3:**
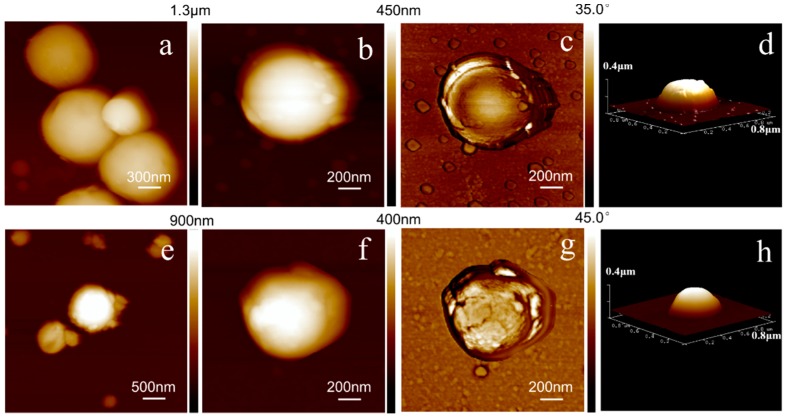
AFM images of the electrosprayed lignin nanospheres and Cu_2_O@L: (**a**,**b**) height map images of the lignin nanospheres; (**c**) phase diagram of the electrosprayed lignin nanosphere; (**d**) 3D image of the electrosprayed lignin nanosphere; (**e**,**f**) height map images of Cu_2_O@L; (**g**) phase diagram of Cu_2_O@L; (**h**) 3D image of Cu_2_O@L.

As shown in Figure 3, height map images of AFM demonstrate the spherical shape of the electrosprayed lignin nanospheres (Figure 3a,b) and Cu_2_O@L (Figure 3e,f). Figure 3c shows the defective surface of the lignin nanosphere, which may contribute to it being a favorable catalyst support for preparing the high-performance lignin-based catalyst. There are two phases of Cu_2_O@L (Figure 3g), indicating Cu_2_O being successfully loaded onto the lignin nanospheres. Besides, the 3D images of AFM (Figure 3d,h) also prove the spherical structures of the lignin nanospheres and Cu_2_O@L. The catalyst Cu_2_O@L exhibits a nanoscale particle size mainly ranging from 100 to 1000 nm, which appears as a large peak at a size of around 600 nm (Figure S2). The particle-size analysis is in accordance with SEM, TEM, and the AFM results mentioned above.

**Figure 4:**
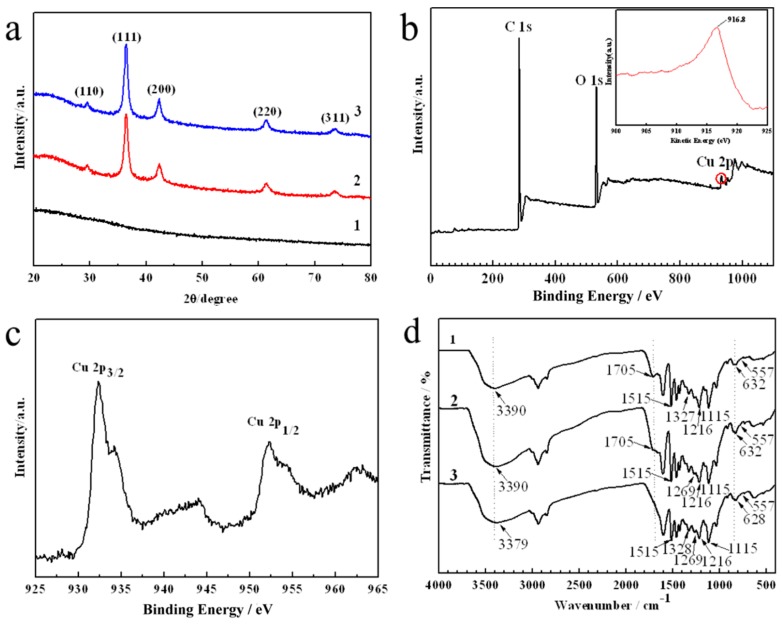
The XRD, XPS, Cu L3VV signal, and FTIR spectra of the samples: (**a**) the XRD of lignin nanospheres and Cu_2_O@L: (a-1) XRD of lignin nanospheres, (a-2) Cu_2_O@L, and (a-3) fourth-time-reused Cu_2_O@L; (**b**) the XPS and Cu L3VV signal of Cu_2_O@L; (**c**) Cu 2p XPS spectrum peaks of Cu_2_O@L; (**d**) the FTIR spectra of lignin and lignin nanosphere-based catalyst: (d-1) raw lignin, (d-2) lignin nanospheres, and (d-3) Cu_2_O@L.

The crystal structure of Cu_2_O@L was confirmed by XRD patterns. As shown in Figure 4a-1, the dispersion peak at 2*θ* = 30–40° is a typical XRD diffraction pattern of noncrystalline lignin. Because the overlap peak of the lignin diffraction peaks heighten at 29°, 36°, and 42° of Cu_2_O [43], all of the sharp diffraction peaks (2*θ* = 29°, 36°, 42°, 62°, and 74°) can be indexed to the face-centered cubic structure of Cu_2_O (JCPDS file: 65-3288) (Figure 4a-2). There are still XRD diffraction peaks of Cu_2_O from the reused Cu_2_O@L (Figure 4a-3). The XRD results are consistent with the results of HRTEM, indicating the successful loading of Cu_2_O onto the lignin nanospheres.

XPS and Cu L3VV Auger line results for Cu_2_O@L were studied to investigate the components of the catalyst and the chemical state of the Cu element. The XPS complete spectra shows C 1s, O 1s, and Cu 2p peaks (Figure 4b). The result illustrates that there are C, O, and Cu elements in Cu_2_O@L, corresponding with the EDS result. The binding energies of the Cu 2p of Cu_2_O@L are located at 932.5 and 952.5 eV. In addition, there is a satellite peak at 944.6 eV in the XPS spectrum of the Cu 2p of the Cu_2_O@L (Figure 4c). Furthermore, it is found that the peak of the Cu L3VV signals is centered at 916.8 eV, indicating the primary existence of Cu(I) in the catalyst [44]. According to the HRTEM, XRD, and XPS results, the nanoparticles on electrosprayed lignin nanospheres are calculated to be Cu_2_O.

Moreover, the structural characteristics of Cu_2_O@L were investigated by FTIR spectrum. Figure 4d-1 shows the FTIR spectra of lignin. The characteristic FTIR absorption peaks of alkali lignin (from poplar) are 1327 cm^−1^, 1269 cm^−1^, and 1115 cm^−1^ that are related to guaiacyls, the stretching vibrations of carbon–oxygen bonds, and ether bonds, respectively. The peaks at 3390 cm^−1^, 1216 cm^−1^, 1515 cm^−1^, and 557 cm^−1^ are attributed to phenolic hydroxyl groups, carbon double bonds of benzene, and acyl-oxygen bonds, respectively [45]. As shown in Figure 4d-2, the FTIR spectrum of the electrosprayed lignin nanospheres is similar to that of lignin. Figure 4d-3 shows the FTIR spectrum of Cu_2_O@L. The peak at 3390 cm^−1^ shifts to 3379 cm^−1^, and the peak at 1216 cm^−1^ weakens, indicating interaction of phenolic hydroxyl and cuprous groups. As compared with lignin and lignin nanospheres, the peak at 1705 cm^−1^ disappears in the FTIR spectrum of Cu_2_O@L. This was probably due to the weakened π bond of carbon and oxygen resulting from interaction of cuprous and carbon–oxygen bonds. There are also some differences in the fingerprint region. The characteristic peak at 628 cm^−1^ may be attributed to Cu_2_O, which is caused by the overlap of the peak the 632 cm^−1^ from lignin and the peak of 624 cm^−1^ from Cu_2_O.

Furthermore, thermal stabilities of the lignin nanospheres and Cu_2_O@L were displayed in Figure S3. The lignin nanospheres and the catalyst have similar thermal properties, in that both of them show decomposition temperatures above 150 °C. Because the Huisgen [3+2] cycloaddition reactions in this study were carried out at room temperature, the thermal properties of Cu_2_O@L could fully meet the temperature requirement of the reaction.

### 3.2. Catalytic Performance of Lignin Nanosphere-Supported Catalyst in the Huisgen “Click” Reaction

Solvent-free conditions confer to the reaction process simple operation, easy separation, and reduced environmental hazards [46]. With the aim to develop a “green” method for “click” chemistry, we attempted to synthetize triazoles under solvent-free conditions. The result is inspiring. As shown in Table S1, the reaction yield is almost 99% under solvent-free conditions without adding any base (with 1.2 mol % catalyst). The reason for this is that Cu_2_O@L may achieve better contact with reaction reagents in such a situation. The product is able to crystallize out from the reactants during the reaction process, and the whole reaction can be monitored visually under the solvent-free condition. Moreover, different solvents, such as water, alcohol and, methanol have also been adopted as solvents for the reaction of benzyl azide and phenylacetylene. As compared with the solvent-free condition, the reaction yields are slightly lower than those of the other solvents using Cu_2_O@L.

The classic cycloaddition reaction of benzyl azide and phenylacetylene was used to explore the optimum condition of the reaction. The results were encouraging, noting that the reaction between benzyl azide and phenylacetylene could be completed within 3 h under the solvent-free condition at ambient temperature. The effect of the Cu_2_O@L quantity on the reaction was investigated. The reaction almost did not take place under the solvent-free condition at room temperature even after 24 h of stirring without the catalyst. When 0.6 mol % Cu_2_O@L was used, the productivity reached 93%. The reaction could be completed in 3 h and provided a quantitative yield of the desired product with Cu_2_O@L. The optimized result (99% yield) was obtained within 3 h under the solvent-free condition at room temperature with 1.2 mol % Cu_2_O@L, while the yield of the reaction was only 27% and 25% using equivalent Cu_2_O nanoparticles as the catalyst under the solvent-free condition or using H_2_O as the solvent, respectively. Besides, the reaction did not take place when the lignin nanospheres were used alone.

After establishment of the optimum condition for the cycloaddition of benzyl azide and phenyl acetylene, a series of azides and terminal alkynes were further subjected to the Huisgen “click” reactions using Cu_2_O@L at room temperature under the solvent-free condition to further evaluate the efficiency of this methodology. The products obtained have been tabulated in Table 1. All the reactions could be completed within 3 h to give high yields of the corresponding 1,4-triazoles. The substituents of phenyl acetylene (electron-withdrawing, electron-rich, and heterocyclic) had no effect on the reaction, and tolerance for variations in the azide component was also excellent. In particular, the catalyst was very effective for aliphatic alkynes as well, and we could isolate 97% of the product between 4-nitrobenzyl azide and 1-hexyne.

**Scheme 1:**
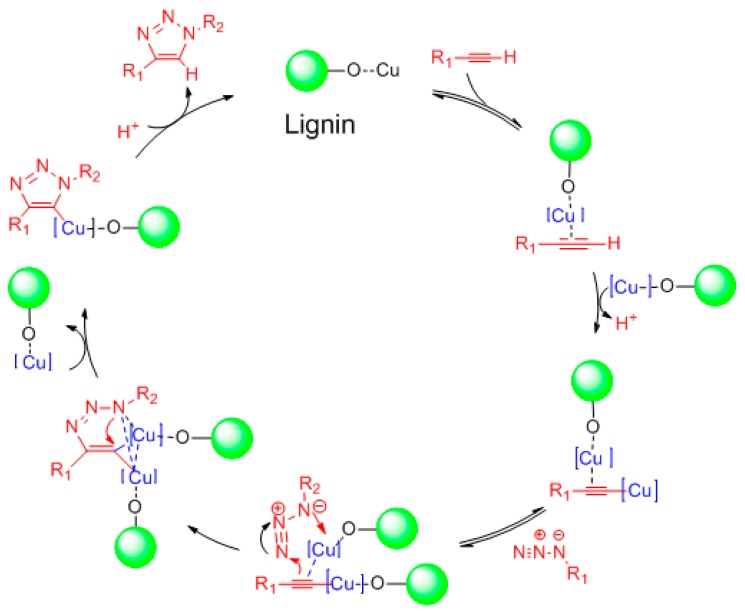
The proposed catalytic mechanism for the “click” reaction catalyzed by Cu_2_O@L (red colors are from reactants and green colors are from catalyst).

The possible mechanism for the catalytic cycle is proposed in Scheme 1, in which the whole reaction process is related to two copper atoms. In our work, Cu(I) is also considered to be the most active species. Cu(I) coordinates with strongly polar phenolic hydroxyl groups in lignin molecules and can form Cu_2_O@L coordination compounds. Then, there is the formation of Cu_2_O@L–acetylidine composites by initial coordination with alkynes. The Cu_2_O@L–acetylidine composites are added to azide groups to form the active cycloaddition composites. Finally, the cycloaddition composites give 1,2,3-triazole.

## 4. Discussion

Cu(I) has always been considered to be one of the most active species in “click” chemistry. In this work, the catalytic active center of Cu_2_O@L is monovalent copper. Furthermore, the lignin nanosphere support has improved the catalytic activity of the Cu_2_O nanoparticles for the yield of Huisgen “click” reactions, attributed to the formation of Cu_2_O@L composites (Figure S4). Therefore, the lignin nanosphere support can further provide sufficient catalytic active sites, which favors the continuation of the azide–alkyne cycloaddition reaction. These may be the reasons why all the yields of the products are impressive under the solvent-free condition at room temperature using Cu_2_O@L. A comparison of some selected protocols in the literature and our method are listed in Table 2. The catalyst system was also characterized by electrochemistry (Figure S5).

Cu_2_O@L could be reused for four times without obvious change in its activity. As shown in Figure S6a, it can be observed that with the increasing number of cycles of the reaction, the catalytic activity of the catalyst does not decrease significantly. The Cu_2_O@L works extremely well even up to four subsequent cycles with 95% yield. These observations suggest that after few cycles, the catalytic efficiency can be preserved, which is attributed to the stable structure of Cu_2_O@L. Metal leaching was studied by AAS analysis of the catalyst before and after four reaction cycles. The Cu element concentration is found to be 7.76% before the reaction and 7.62% after the reaction. The XRD result shows that there are still diffraction peaks in the reused catalyst (Figure 4a-3). The morphology of Cu_2_O@L after four cycles was investigated as well, as shown in Figure S6b,c. It is clearly shown that the Cu_2_O nanoparticles still remain irregular in shape and dispersed uniformly on the surface of the lignin nanospheres. The XRD and TEM results indicate that the lignin nanosphere is a favorable support for cuprous oxide to catalyze Huisgen [3+2] cycloaddition reactions.

## 5. Conclusions

In summary, we developed a facile and powerful method to prepare Cu_2_O@L using widely available and sustainable lignin by electrospraying technology. Alkali lignin was successfully processed into uniform nanospheres when electrospraying lignin precursor solution at an appropriate concentration under a determined voltage condition. The lignin nanosphere was further exploited as part of a high-performance cuprous oxide heterogeneous catalyst, and the catalyst exhibited an excellent catalytic activity for the Huisgen “click” reaction under the solvent-free and mild conditions. As compared with other copper-mediated “click” reactions, reactions adopting the Cu_2_O@L showed higher yield (up to 99%) and required shorter reaction time and relatively mild conditions to synthesize 1,2,3-triazoles. The preparation process of the catalyst is economical, facile, and can be implemented on a large scale. In general, this work provided a “green” method for “click” chemistry and an efficient approach for application of the biomass resource lignin.

## Figures and Tables

**Table 1:** Huisgen “click” reactions using Cu_2_O@L.

Reaction	Alkyl Halide	Alkyne	Product ^a,b^	Yield (%) ^c^
1	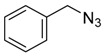	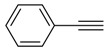	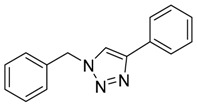	99
2	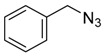	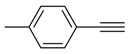	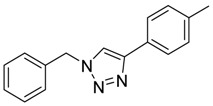	98
3	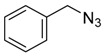	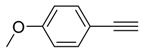	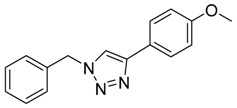	98
4	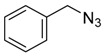	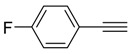	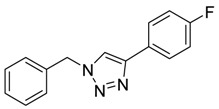	98
5	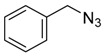	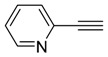	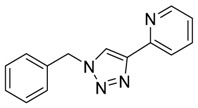	99
6	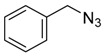	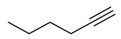	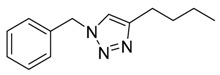	98
7	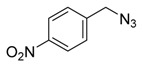	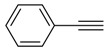	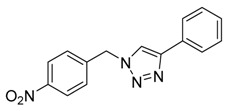	97
8	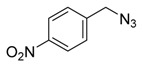	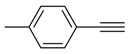	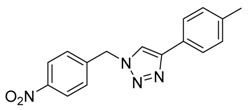	96
9	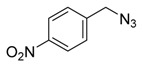	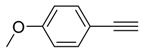	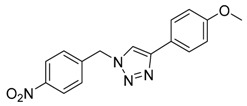	99
10	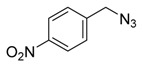	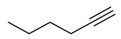	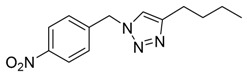	97
11	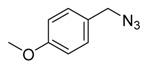	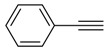	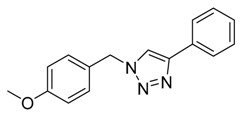	98
12	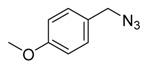	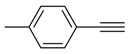	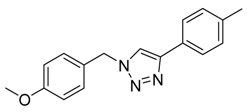	98
13	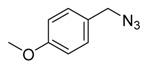	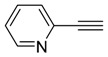	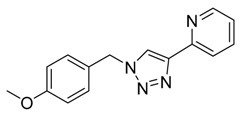	99
14	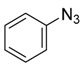	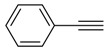	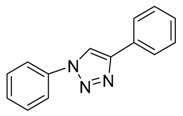	96
15	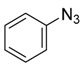	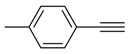	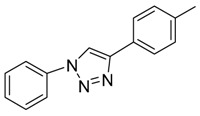	98
16	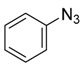	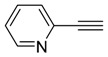	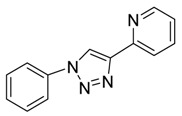	99

^a^ Reaction conditions: 1.0 mmol of alkyl azide, 1.2 mmol of alkyne, 1.2 mol % of Cu_2_O@L, solvent-free, room temperature, 3 h, under air condition. ^b^ Products were characterized using NMR and compared with the reported data. ^c^ Isolated yield, according to mol of alkyl azide.

**Table 2:** The brief comparison of this catalyst with previous ones for Huisgen “click” reactions of azides and alkynes.

Entry	Catalyst	mol % ^a^	Solvent	T (°C)	Time (h)	Yield (%)	Ref.
1	Copper in charcoal	5	dioxane	25	10	84	[47]
2	Copper in zeolite	10	toluene	25	15	94	[48]
3	CuSO_4_·5H_2_O	1	H_2_O/t-BuOH	90	8	89	[49]
4	Photocatalytic-Cu(II)	5	ethanol	25	5	96	[50]
5	Dendrimer-CuI	0.5	H_2_O	25	3	98	[51]
6	Chitosan-CuSO_4_	0.4	H_2_O	25	4–6	99	[8]
7	Cu on chelated resins	1	solvent-free	50/80	24/4	100	[52]
8	CuCl/microwaves	2.5	glycerol	100	0.5	82	[53]
9	CuOAc/ball-milling	10	solvent-free	-	1	90	[54]
10	CuO@Nb_2_O_5_/UV	1–1.2	THF	25	6	99	[55]
11	Cu_2_O Nanocrystals	6.25	EtOH	55	1	96	[56]
12	Cu_2_O@L	1.2	solvent-free	25	3	99	This work

^a^ Catalyst additive amount, mol %.

## Supplementary Materials

The following are available online at http://www.mdpi.com/2073-4360/10/7/724/s1, Figure S1: SEM images of electrosprayed lignin from various concentrations of lignin solutions: (a) 25%, (b) 35%, (c) 45%, and (d) 55%, respectively; Figure S2: Particle size distribution of Cu2O@L; Figure S3: The TGA of lignin and Cu2O@L: (a) raw lignin, (b) lignin nanospheres, and (c) Cu2O@L, respectively; Figure S4: The XPS spectrum of lignin nanospheres and Cu2O@L: (a) lignin nanospheres, (b) Cu2O@L, (c) O 1s XPS peaks of lignin nanospheres, (d) O1s XPS peaks of Cu2O@L, (e) C 1S XPS peaks of lignin nanospheres, (f) C 1S XPS peaks of Cu2O@L, (g) Cu 2p XPS spectrum of Cu2O nanoparticles, (h) Cu2p XPS spectrum of Cu2O@L, (i) XPS spectrum and Cu L3VV Auger line of lignin nanospheres as a reductant to prepare Cu2O@Lignin, respectively; Figure S5: Recycling experiment and TEM images of Cu2O@L: (a) Recycling experiment of Cu2O@L, (b) fresh Cu2O@L, and (c) the fourth-time-recycled Cu2O@L, respectively; Figure S6: Recycling experiment and TEM images of Cu2O@L: (a) Recycling experiment of Cu2O@L, (b) fresh Cu2O@L, and (c) the fourth-time-recycled Cu2O@L, respectively; Scheme S1. Chemical equation of preparing Cu2O@Lignin with lignin as an assistant reducing agent; Table S1. Effect of polarity of solvent on Huisgen “click” reaction of azides and alkynes using Cu2O@L in different solvents; Spectroscopic data, 1H and 13C NMR Spectra of products.

## Abbreviations

XRD	X-ray powder diffraction
SEM	Scanning electron microscopy
TEM	Transmission electron microscopy
XPS	X-ray photoelectron spectroscopy
AFM	Atomic force microscope
LSCM	Laser scanning confocal microscope
EDS	Energy dispersive analysis system of X-ray
TGA	Thermogravimetric analysis
FT-NMR	Fourier transform nuclear magnetic resonance
UV–vis–NIR	UV-visible-near infrared spectroscopy
FTIR	Flourier transformation infrared spectroscopy
AAS	Atomic absorption spectrophotometry
Cu_2_O@L	the lignin nanosphere-supported cuprous oxide
DMF	*N*,*N*-dimethylformamide
ID	inner diameter
FL	Fluorescence spectra
PMT	Photomultiplier tube
M_W_	weight-average molecular weight
TMS	tetramethylsilane
