# Supplementary material for: Lignin Nanosphere-Supported Cuprous Oxide as an Efficient Catalyst for Huisgen [3+2] Cycloadditions under Relatively Mild Conditions
Source: Polymers (Basel). 2018 Jul 2;10(7):724. doi: 10.3390/polym10070724 (PMC6403750; doi:10.3390/polym10070724)
Supplement: Supplementary file 1 [file polymers-10-00724-s001.pdf]

Lignin Nanosphere-Supported Cuprous Oxide as an Efficient Catalyst for Huisgen [3+2] Cycloadditions under Relatively Mild Conditions

Zidan Zhou [1], Xinwen Peng[1,*], Linxin Zhong[1,*], Xuehui Li [2], and Runcang Sun [3]

[1] State Key Laboratory of Pulp and Paper Engineering, South China University of Technology, Guangzhou 510641, China;
[2] School of Chemistry and Chemical Engineering, South China University of Technology, Guangzhou 510641, China;
[3] Beijing Key Laboratory of Lignocellulosic Chemistry, Beijing Forestry University, Beijing 100083, China.
* Correspondence: fexwpeng@scut.edu.cn or lxzhong0611@scut.edu.cn

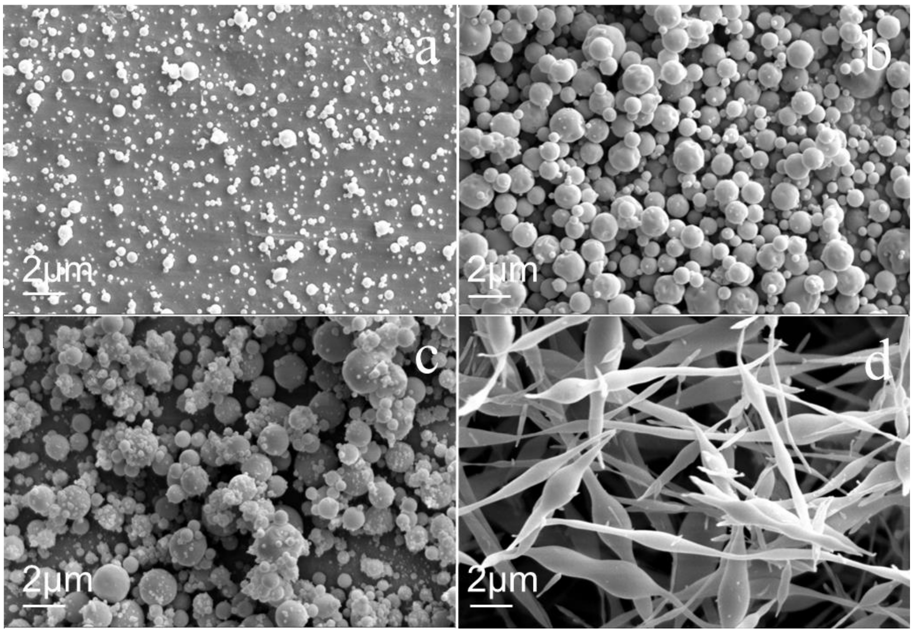

Figure S1. SEM images of electrosprayed lignin from various concentrations of lignin solutions: (a) 25%, (b) 35%, (c) 45%, and (d) 55%, respectively.

The catalyst Cu_2O@L shows a regular spherical morphology (see SEM and TEM results), with diameter and exhibits a nanoscale particle size mainly ranging from 100 to 1000 nm, which is with the appearance of a large peak at a size of around 600 nm, as shown in Figure S2. The particle-size analysis is in accordance with the SEM, TEM, and AFM results above.

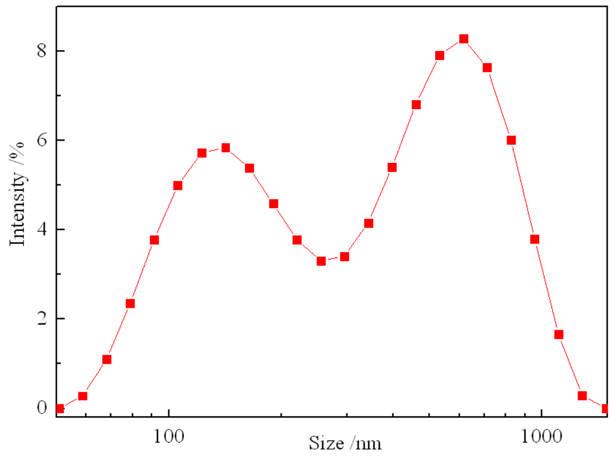

Figure S2. Particle size distribution of Cu_2O@L (emphasizing with red color).

Thermal stabilities of the lignin nanospheres and Cu_2O@L are displayed in Figure S3. The lignin nanospheres and the catalyst have similar thermal properties, in that both of them show decomposition temperatures above 150 °C. Since the Huisgen [3+2] cycloaddition reactions in this study were carried out at room temperature, the thermal properties of Cu_2O@L could fully meet temperature requirement of the reaction.

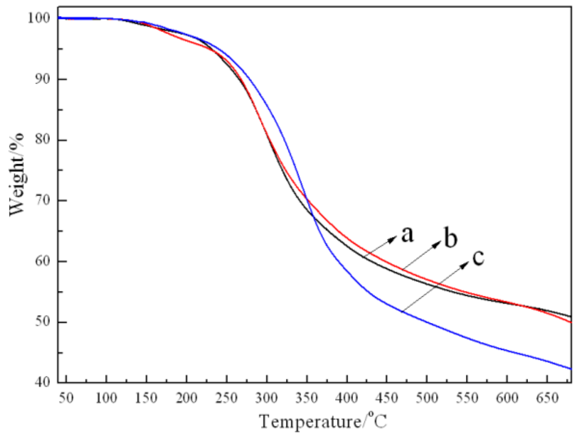

Figure S3. The TGA of lignin and Cu_2O@L: (a) raw lignin, (b) lignin nanospheres, and (c) Cu_2O@L, respectively.

Table S1. Effect of solvent polarity on the Huisgen "click" reaction of azides and alkynes, using Cu_2O@L in different solvents.

Entry	Solvent (Ratio[b])	Yield[c]
1	Solvent-free	99
2	H_2O	87
3	C_2H_5OH	99
4	C_4H_9OH	98
5	H_2O/C_2H_5OH (1:1)	87
6	H_2O/C_4H_9OH (1:1)	73

[a] Reaction conditions: 1.0 mmol of alkyl azide, 1.2 mmol of alkyne, 1.2 mol % of Cu_2O@L, at room temperature, 3 h, under air condition. [b] volume ratio, in 4 ml solvent. [c] Isolated yield, according to mol of alkyl azide.

The oxygen atoms of lignin molecules are directly involved in the interactions with cuprous groups by the valence bond. As presented in Figure S4(a) and Figure S4(b), there are C and O elements in the XPS spectrum of the lignin, while the three elements of C, O, and Cu appear in the XPS spectrum of Cu_2O@L. The Cu L3VV Auger line is studied to investigate the chemical state of the Cu element, indicating the existence of Cu(I) in the catalyst. As shown in Figure S4(c) and Figure S4(d), the coordination of copper to lignin is clearly reflected by the remarkable changes of the O1s peak of Cu_2O@L. Besides the two components observed in the spectrum of lignin, there is a peak corresponding to Cu–O–Cu centered at 530.8 eV, and a new component appeared at 533.1 eV in Figure S4(d). According to the previous literature, the new component may be a result of the formation of C–O–Cu coordination bond. As shown in Figure S4e and Figure S4f, the components in Cu_2O@L (284.6eV and 286.0eV) were similar to those of the lignin nanospheres (284.6eV and 286.2eV), while there was an another peak at 289.2 eV compared with the C1s signal of the lignin nanospheres. This indicated that the electronic situation of carbon atoms was influenced by the binding effect between the lignin nanospheres and Cu_2O. This fact is also revealed by a shift of the Cu 2p peaks of copper. The binding energies of Cu 2p of Cu_2O@L composites are located at 932.5 and 952.5 eV, which are lower than that of the copper element at 933.0 and 953.1 eV in Cu_2O nanoparticles excited by Mg Kα, as shown in Figure S4(g) and Figure S4(h). The shifts of the binding energies of oxygen and copper are probably due to the transference of electrons from oxygen atoms in lignin molecules to copper, which results in the increase of the negative charges of copper and the decrease of binding energy. On the contrary, the binding energy of the corresponding oxygen atoms in C–O groups of Cu_2O@L composites increases from 531.2 eV to 531.6 eV. Meanwhile, the area of the component located at 532.6 eV significantly decreases (Figure S4(c) and Figure S4(d)). Accordingly, we could summarize that copper atoms mainly coordinated with phenolic hydroxyl groups in lignin and were presented in the C–O–Cu form in Cu_2O@L. The XPS results are consistent with the FTIR spectrum results stated above. Furthmore, XPS spectra of lignin nanospheres as a reductant to prepare Cu_2O@Lignin and the Cu L3VV Auger line were also studied to investigate the chemical state of the Cu element. As shown in Figure S4(i), it is a Cu L3VV Auger line of the cuprous groups of the sample (Cu_2O@Lignin) that was prepared without adding other reductants (Scheme S1).

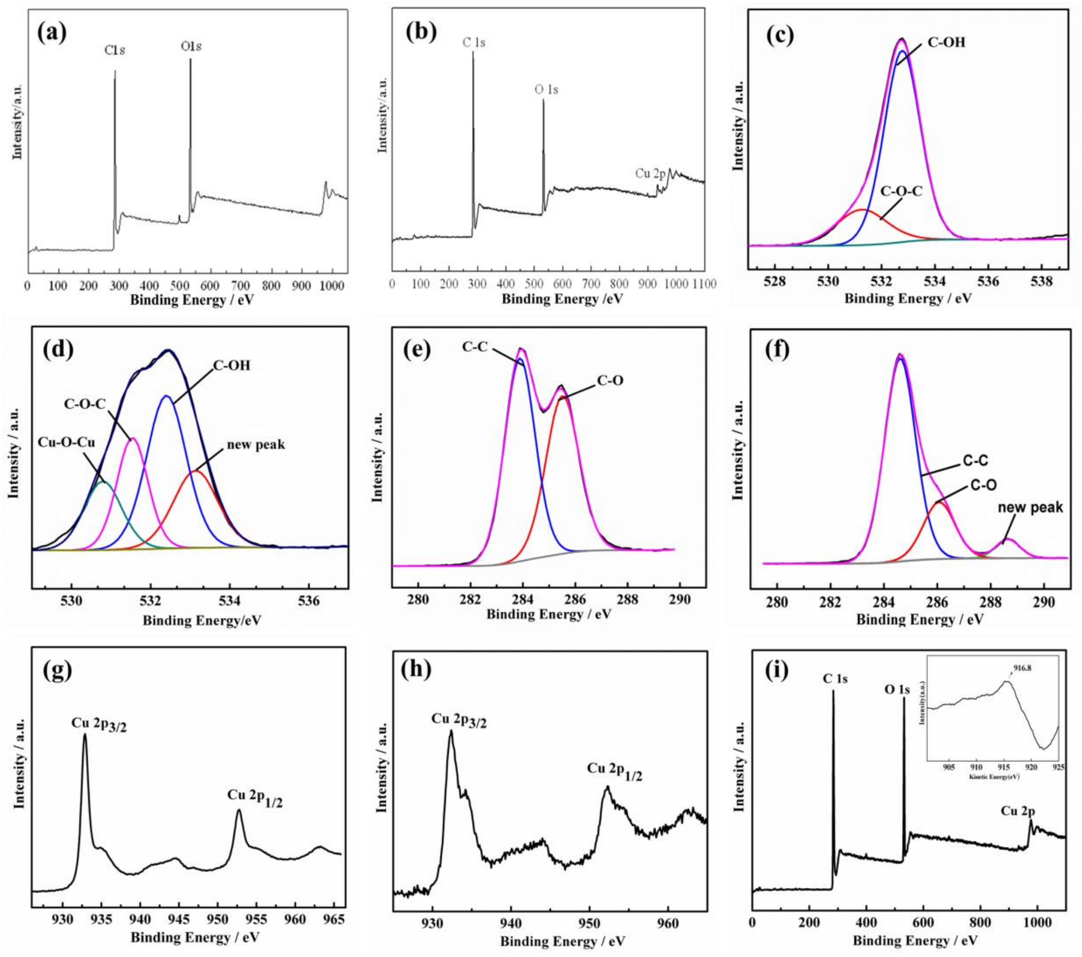

Figure S4. The XPS spectrum of lignin nanospheres and Cu_2O@L: (**a**) lignin nanospheres, (**b**) Cu_2O@L, (**c**) O1s XPS peaks of lignin nanospheres, (**d**) O1s XPS peaks of Cu_2O@L, (**e**) C1s XPS peaks of lignin nanospheres, (**f**) C1s XPS peaks of Cu_2O@L, (**g**) Cu2p XPS spectrum of Cu_2O nanoparticles, (**h**) Cu2p XPS spectrum of Cu_2O@L, (**i**) XPS spectrum and Cu L3VV Auger line of lignin nanospheres as a reductant to prepare Cu_2O@Lignin.

A similar preparation process was also carried out without using the reducing agent hydrazine hydrate to demonstrate the reducing property of the lignin, and the sample was dried to obtain Cu_2O@Lignin. The Cu element content in this Cu_2O@Lignin sample is 1.4% (w/w, confirmed by AAS analysis). The results can indicate that the lignin nanopheres possessed the reduction property to deoxidize copper sulfate. The yield of the reaction between benzyl azide and phenylacetylene is 96% using Cu_2O@Lignin as the catalyst, within 6 h, under the solvent-free condition, at ambient temperature.

In addition, the catalyst systems were characterized by electrochemistry. The result showed that the Cu_2O@L was a stable catalyst, because there were no oxygen peaks in the cyclic voltammetry curve of the catalyst system, as shown in the following Figure S5.

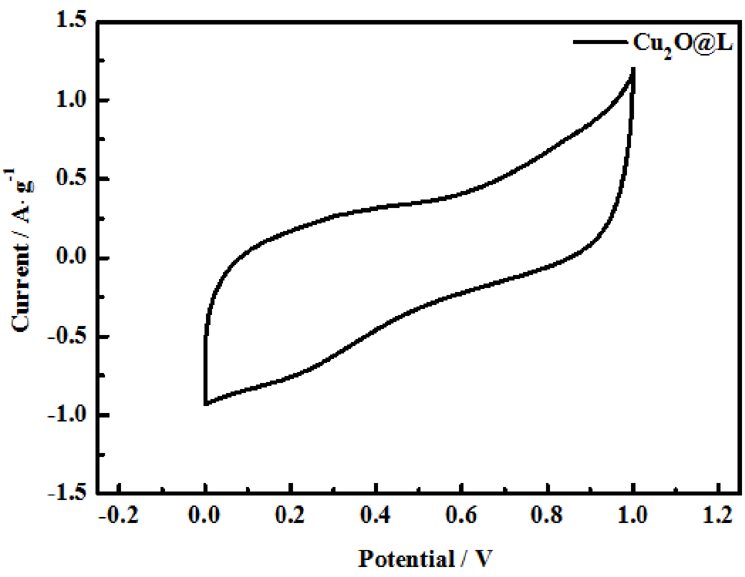

Figure S5. Cyclic voltammetry curve of the Cu_2O@L catalyst in 1 M Na_2SO_4.

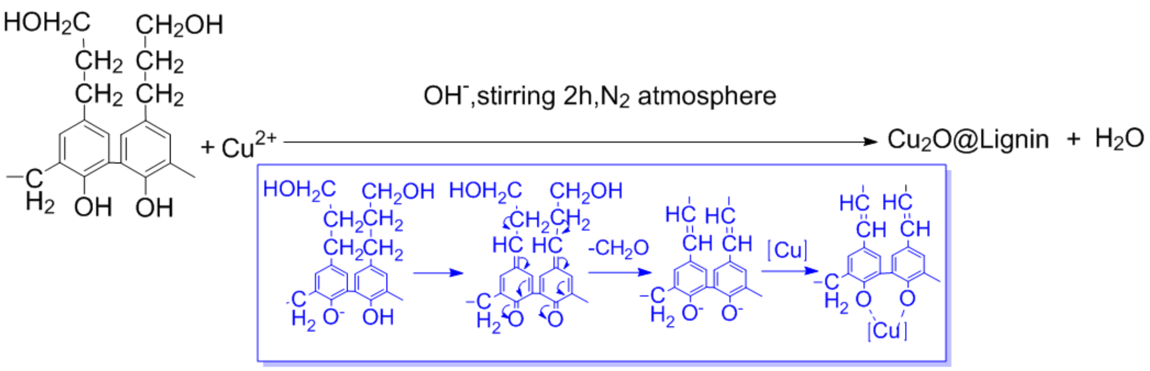

Scheme S1. Chemical equation of preparing Cu_2O@Lignin with lignin as an assistant reducing agent.

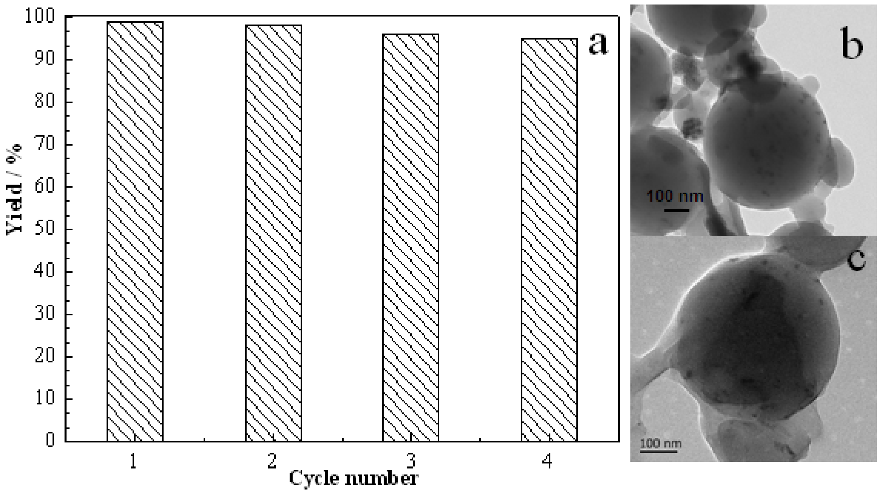

Figure S6. Recycling experiment and TEM images of Cu_2O@L: (a) Recycling experiment of Cu_2O@L, (b) fresh Cu_2O@L, and (c) the fourth-time-recycled Cu_2O@L, respectively.

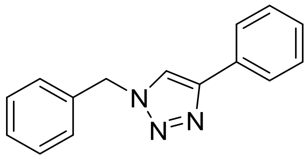

1-Benzyl-4-phenyl-1H-1,2,3-triazole: ^{1}H NMR (600 MHz, CDCl3) δ 7.78 (d, J = 6.0 Hz , 2H), 7.66 (s, 1H), 7.38-7.24 (m, 8H), 5.53 (s, 2H); ^{13}C NMR (151 MHz, CDCl3): δ (ppm) 148.33, 134.73, 130.58, 129.11, 128.78, 128.72, 128.12, 128.01, 125.68, 119.59, 54.15.

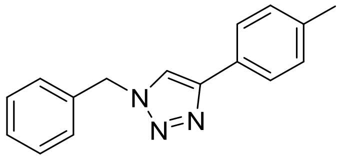

1-Benzyl-4-(p-Tolyl)-1H-1,2,3-triazole: ^{1}H NMR (600 MHz, CDCl3) δ 7.68 (d, J = 6.0 Hz, 2H), 7.61 (s, 1H), 7.38-7.34 (m, 3H), 7.29-7.28 (m, 2H), 7.19 (d, J = 6.0 Hz, 2H), 5.54 (s, 2H), 2.35 (s, 3H). ^{13}C NMR (151 MHz, CDCl3): δ (ppm) 148.30, 137.98, 134.79, 129.47, 129.13, 128.76, 128.05, 127.76, 125.62, 119.17, 54.18, 21.26.

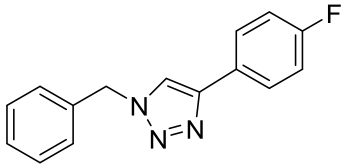

1-Benzyl-4-(4-Fluorophenyl)-1H-1,2,3-triazole: ^{1}H NMR (600 MHz, CDCl3) δ 7.75-7.73 (m, 2H), 7.65 (s, 1H), 7.36-7.26 (m, 5H), 7.05 (t, J = 6.0 Hz, 2H), 5.52 (s, 2H). ^{13}C NMR (151 MHz, CDCl3): δ (ppm) 163.44, 161.80, 147.29, 134.67, 129.14, 128.78, 128.04, 127.46, 127.40, 126.86, 126.83, 119.53, 115.82, 115.67, 54.19.

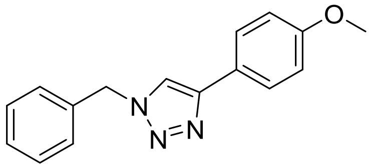

1-Benzyl-4-(4-methoxyphenyl)-1H-1,2,3-triazole: ^{1}H NMR (600 MHz, CDCl3) δ 7.72-7.70 (m, 2H), 7.57 (s, 1H), 7.38-7.28 (m, 3H), 7.29-7.28 (m, 2H), 6.92-6.91 (m, 2H), 5.53 (s, 2H), 3.81 (s, 3H). ^{13}C NMR (151 MHz, CDCl3): δ (ppm) 159.62, 148.08, 134.82, 129.11, 128.71, 128.03, 127.01, 123.32, 118.73, 114.22, 55.30, 54.16.

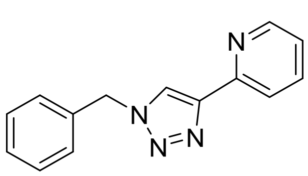

2-(1-Benzyl-1H-1,2,3-triazol-4-yl)Pyridine: ^{1}H NMR (600 MHz, CDCl3) δ 8.52-8.51 (d, J = 6.0 Hz, 1H), 8.17 (d, J = 6.0 Hz, 1H), 8.08 (s, 1H), 7.74 (t, J = 6.0 Hz, 1H), 7.35-7.30 (m, 5H), 7.19-7.17 (t, J = 6.0 Hz, 1H), 5.56 (s, 2H). ^{13}C NMR (151 MHz, CDCl3): δ (ppm) 150.26, 149.33, 148.72, 136.84, 134.41, 129.13, 128.79, 128.26, 122.81, 121.96, 120.18, 54.32.

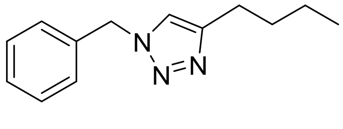

1-Benzyl-4-butyl-1H-1,2,3-triazole: ^{1}H NMR (600 MHz, CDCl3) δ7.38-7.22 (m, 5H), 7.20 (s, 1H), 5.48 (s, 2H), 2.68 (t, J = 6.0 Hz, 2H), 1.65-1.60 (m, 2H), 1.39-1.33

(m, 2H), 0.91 (t, J = 6.0 Hz, 3H). ^{13}C NMR (151 MHz, CDCl3): δ (ppm) 148.90, 135.09, 129.03, 128.56, 127.94, 120.52, 53.93, 31.52, 25.41, 22.31, 13.80.

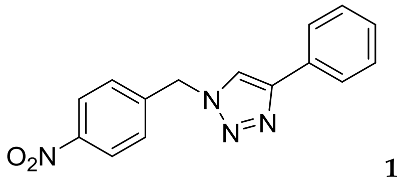

1-(4-Nitrobenzyl)-4-Phenyl-1H-1,2,3-triazole: ^{1}H NMR (600 MHz, CDCl3) δ 8.22-8.21 (m, 2H), 7.81-7.80 (m, 2H), 7.77 (s, 1H), 7.50-7.28 (m, 5H), 5.69 (s, 2H). ^{13}C NMR (151 MHz, CDCl3): δ (ppm) 148.69, 148.11, 141.78, 130.12, 128.93, 128.58, 128.50, 125.77, 124.32, 119.79, 53.19.

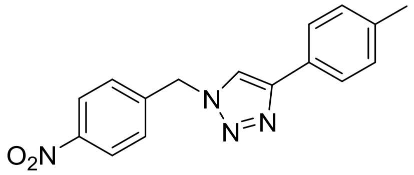

1-(4-Nitrobenzyl)-4-(p-tolyl)-1H-1,2,3-triazole: ^{1}H NMR (600 MHz, CDCl3) δ 8.18-8.17 (d, J = 6.0 Hz, 2H), 7.75 (s, 1H), 7.68 (d, J = 12.0 Hz, 2H), 7.41 (d, J = 12.0 Hz, 2H), 7.20 (d, J = 6.0 Hz, 2H), 5.66 (s, 2H), 2.36 (s, 3H) ^{13}C NMR (151 MHz, CDCl3): δ (ppm) 148.70, 148.01, 141.92, 138.38, 129.59, 128.56, 127.34, 125.65, 124.25, 119.55, 53.09, 21.27.

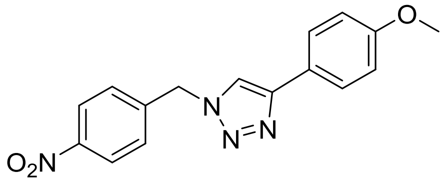

4-(4-Methoxyphenyl)-1-(4-nitrobenzyl)-1H-1,2,3-triazole: ^{1}H NMR (600 MHz, CDCl3) δ 8.20 (d, J = 12.0 Hz, 2H), 7.72 (d, J = 6.0 Hz, 2H), 7.69 (s, 1H), 7.42 (d, J = 12.0 Hz, 2H), 6.93 (d, J = 6.0 Hz, 2H), 5.67 (s, 2H), 3.83 (s, 3H). ^{13}C NMR (151 MHz, CDCl3): δ (ppm) 159.85, 148.55, 148.05, 141.91, 128.55, 127.08, 124.28, 122.84, 118.99, 114.33, 55.34, 53.12.

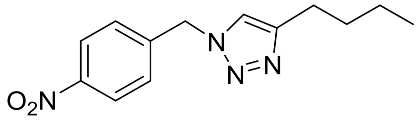

4-Butyl-1-(4-nitrobenzyl)-1H-1,2,3-triazole: ^{1}H NMR (600 MHz, CDCl3) δ 8.20 (d, J = 6.0 Hz, 2H), 7.41 (d, J = 6.0 Hz, 2H), 7.36 (s, 1H), 5.65 (s, 2H), 2.73-2.71 (t, J = 6.0 Hz, 2H), 1.67-1.62 (m, 2H), 1.41-1.34 (m, 2H), 0.92 (t, J = 6.0 Hz, 3H). ^{13}C NMR (151 MHz, CDCl3): δ (ppm) 149.38, 147.94, 142.36, 128.51, 124.19, 121.00, 52.90, 31.45, 25.36, 22.29, 13.79.

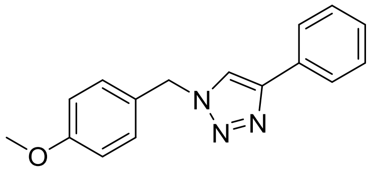

1-(4-Methoxybenzyl)-4-Phenyl-1H-1,2,3-triazole: ^{1}H NMR (600 MHz, CDCl3) δ 7.78-7.77 (m, 2H), 7.62 (s, 1H), 7.38-7.36 (m, 2H), 7.30-7.23 (m, 3H), 6.90-6.87 (m, 2H), 5.47 (s, 2H), 3.78 (s, 3H). ^{13}C NMR (151 MHz, CDCl3): δ (ppm) 159.99, 148.08, 130.63, 129.64, 128.77, 128.09, 126.67, 125.68, 119.35, 114.51, 55.61, 53.74.

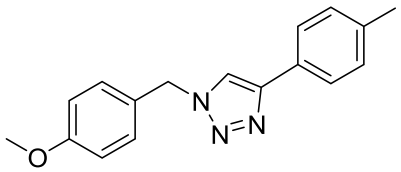

1-(4-Methoxybenzyl)-4-(p-tolyl)-1H-1,2,3-triazole: ^{1}H NMR (600 MHz, CDCl3) δ 7.67 (d, J = 6.0 Hz, 2H), 7.58 (s, 1H), 7.25-7.23 (m, 2H), 7.18 (d, J = 12.0 Hz, 2H), 6.88 (d, J = 6.0 Hz, 2H), 5.46 (s, 2H), 3.78 (s, 3H), 2.34 (s, 3H). ^{13}C NMR (151 MHz, CDCl3): δ (ppm) 159.93, 148.16, 137.90, 129.63, 129.45, 127.83, 126.74, 125.58, 118.97, 114.49, 55.32, 53.70, 21.40.

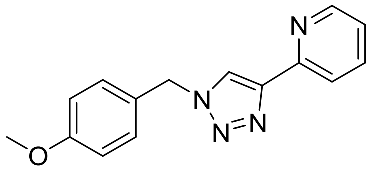

2-(1-(4-Methoxybenzyl)-1H-1,2,3-triazol-4-yl)Pyridine: ^{1}H NMR (600 MHz, CDCl3) δ 8.52-8.51 (m, 1H), 8.16-8.14 (m, 1H), 8.04 (s, 1H), 7.73-7.70 (m, 1H), 7.29-7.22 (m, 2H), 7.17-7.15 (m, 1H), 6.90-6.83 (m, 2H), 5.49 (s, 2H), 3.76 (s, 3H). ^{13}C NMR (151 MHz, CDCl3): δ (ppm) 159.96, 150.30, 149.32, 148.57, 136.82, 129.84, 126.42, 122.76, 121.79, 120.12, 114.50, 55.29, 53.82.

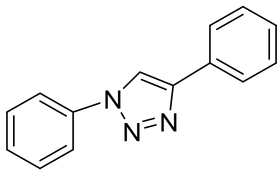

1,4-Diphenyl-1H-1,2,3-triazole: ^{1}H NMR (600 MHz, DMSO-d6) δ 9.31 (s, 1H), 7.98-7.97 (m, 4H), 7.66-7.64 (m, 2H), 7.54-7.50 (m, 3H), 7.41-7.39 (m, 1H). ^{13}C NMR (151 MHz, DMSO-d6): δ (ppm) 147.81, 137.15, 130.76, 130.40, 129.46, 129.19, 128.71, 125.84, 120.50, 120.09.

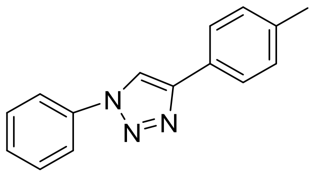

1-Phenyl-4-(p-tolyl)-1H-1,2,3-triazole: ^{1}H NMR (600 MHz, DMSO-d6) δ 9.25 (s, 1H), 7.96 (d, J = 6.0 Hz, 2H), 7.85 (d, J = 6.0 Hz, 2H), 7.65-7.63 (m, 2H), 7.53-7.51 (m, 1H), 7.31 (d, J = 6.0 Hz, 2H), 2.36 (s, 3H). ^{13}C NMR (151 MHz, DMSO-d6): δ (ppm) 147.88, 138.08, 137.18, 130.38, 130.00, 129.11, 127.98, 125.78, 120.44, 119.62, 21.34.

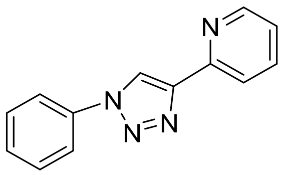

2-(1-Phenyl-1H-1,2,3-triazol-4-yl)Pyridine: ^{1}H NMR (600 MHz, DMSO-d6) δ 9.35 (s, 1H), 8.68-8.67 (m, 1H), 8.15-8.13 (m, 1H), 8.06-8.04 (m, 2H), 7.98-7.95 (m, 1H), 7.65-7.62 (m, 2H), 7.54-7.52 (m, 1H), 7.43-7.41 (m, 1H). ^{13}C NMR (151 MHz, DMSO-d6): δ (ppm) 150.08, 150.04, 148.71, 137.69, 137.06, 130.29, 129.21, 123.71, 121.66, 120.63, 120.27.

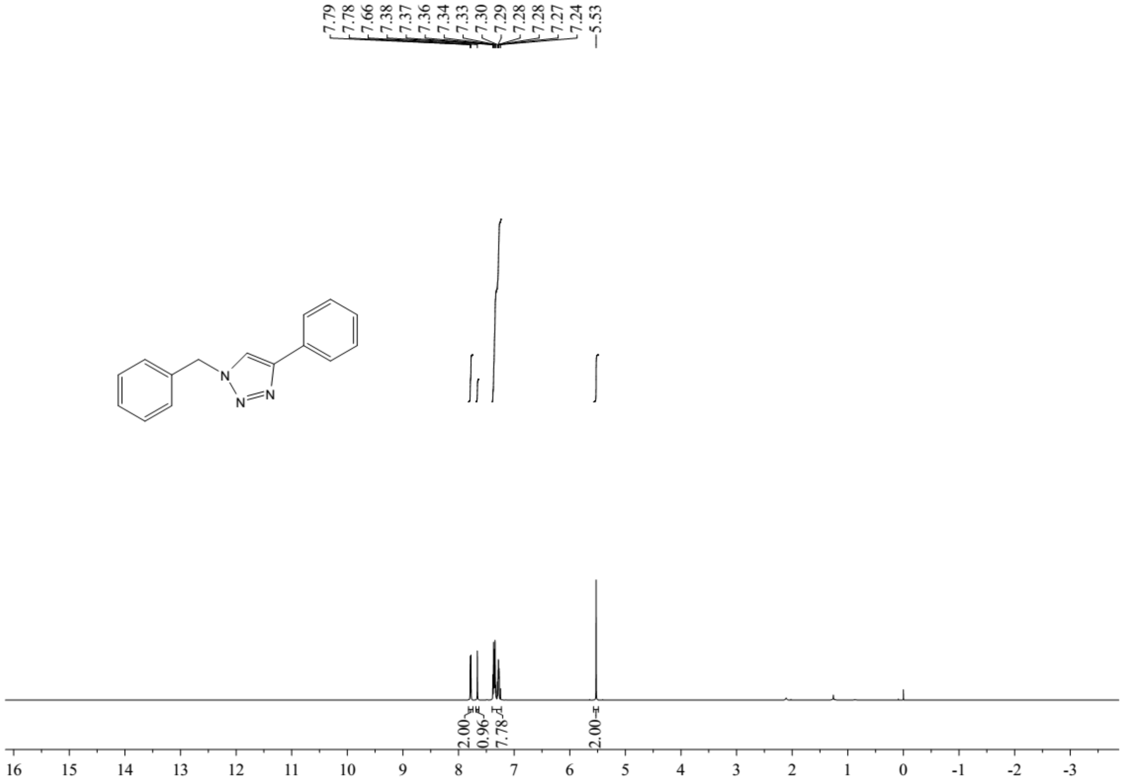
7.79
7.78
7.66
7.38
7.37
7.36
7.34
7.33
7.30
7.29
7.28
7.28
7.27
7.24
5.53
N
N
N
2.00
0.96
7.78
2.00
16 15 14 13 12 11 10 9 8 7 6 5 4 3 2 1 0 -1 -2 -3

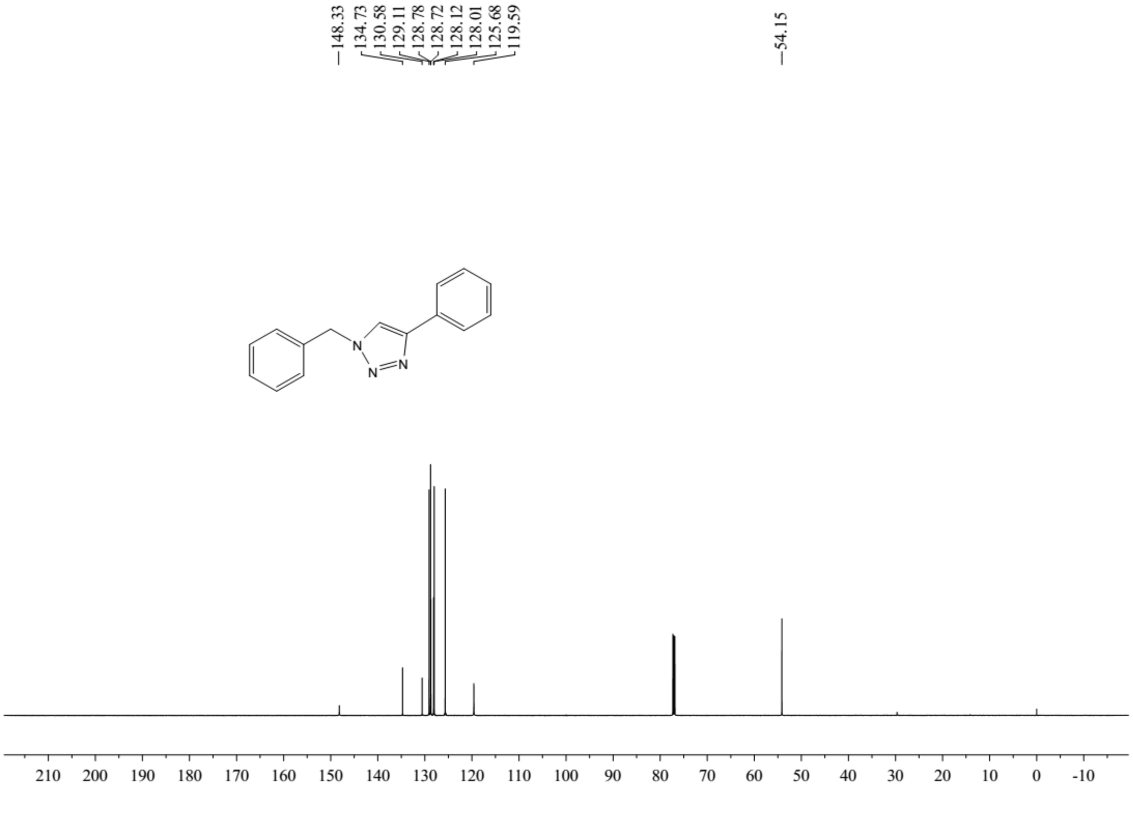
148.33
134.73
130.58
129.11
128.78
128.72
128.12
128.01
125.68
119.59
54.15
N
N
N
210 200 190 180 170 160 150 140 130 120 110 100 90 80 70 60 50 40 30 20 10 0 -10

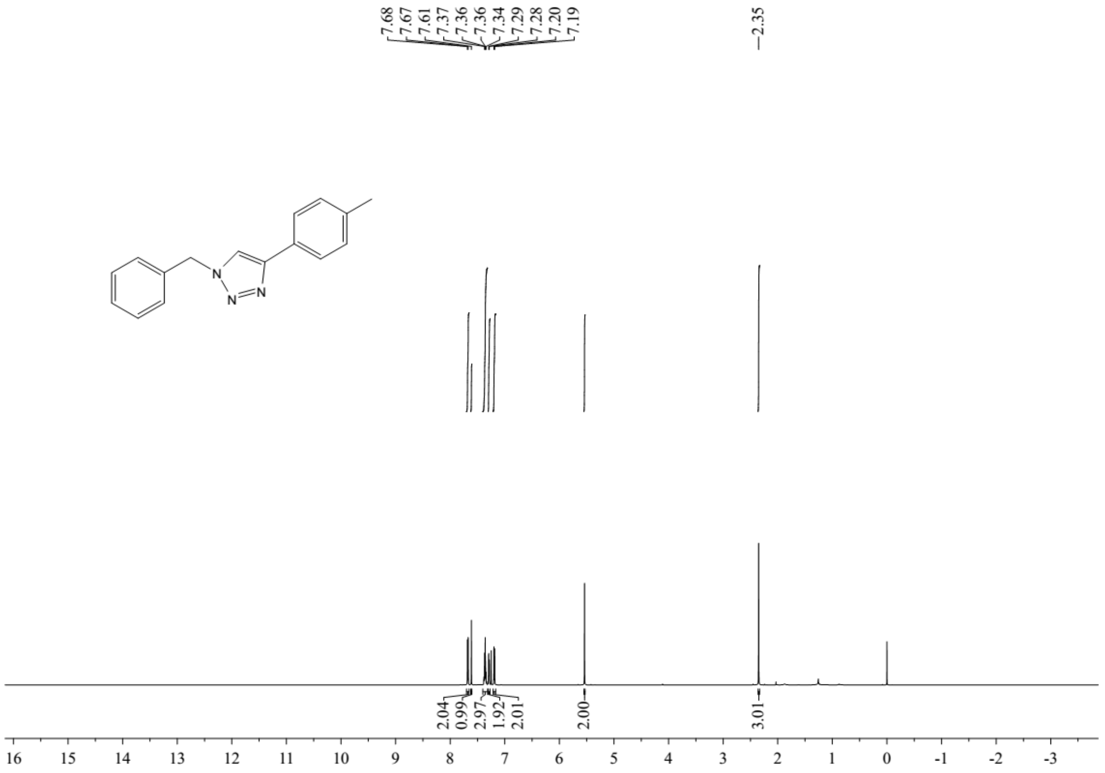
7.68
7.67
7.61
7.37
7.36
7.36
7.34
7.29
7.28
7.20
7.19
2.35
2.04
0.99
2.97
1.92
2.01
2.00
3.01
16 15 14 13 12 11 10 9 8 7 6 5 4 3 2 1 0 -1 -2 -3

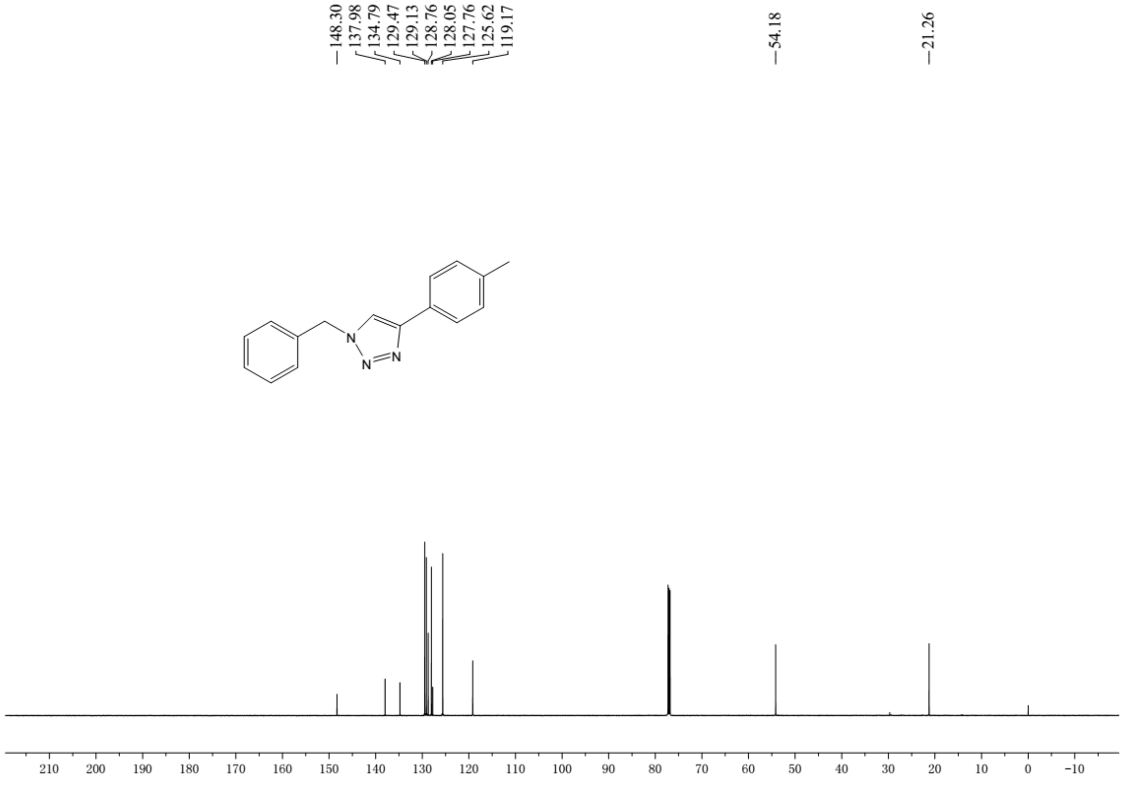
148.30
137.98
134.79
129.47
129.13
128.76
128.05
127.76
125.62
119.17
54.18
21.26
210 200 190 180 170 160 150 140 130 120 110 100 90 80 70 60 50 40 30 20 10 0 −10

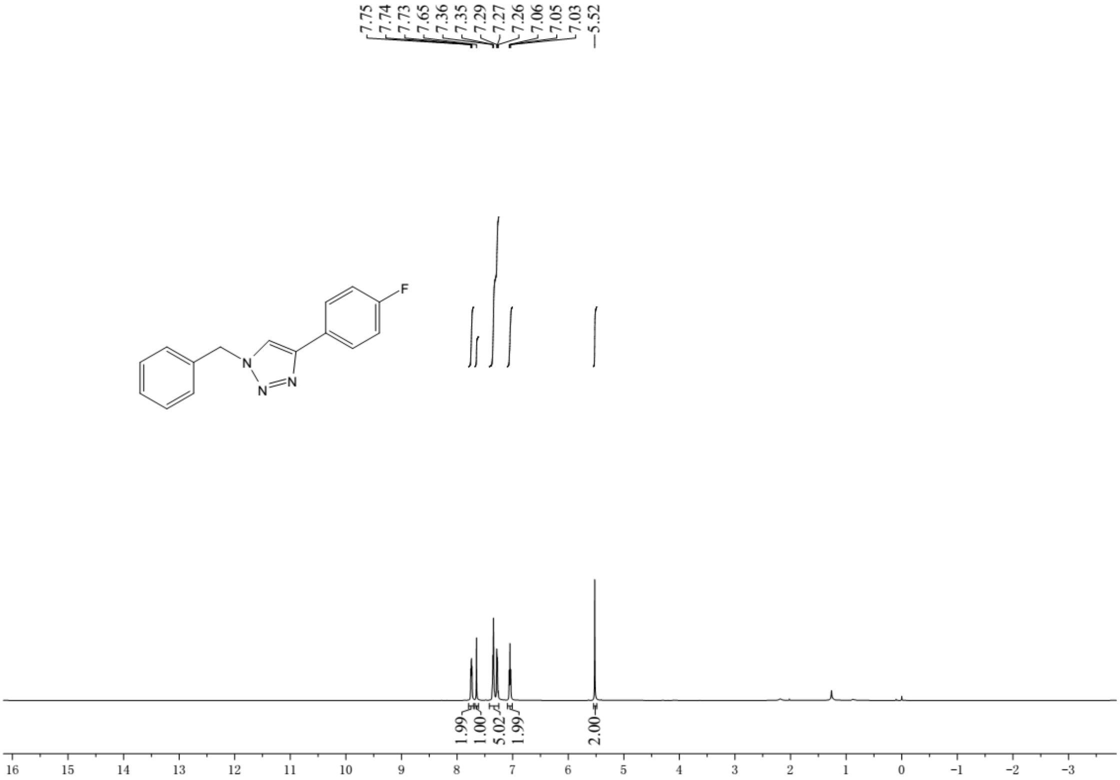
7.75
7.74
7.73
7.65
7.36
7.35
7.29
7.27
7.26
7.06
7.05
7.03
5.52
F
N
N
N
1.99
1.00
5.02
1.99
2.00
16 15 14 13 12 11 10 9 8 7 6 5 4 3 2 1 0 -1 -2 -3

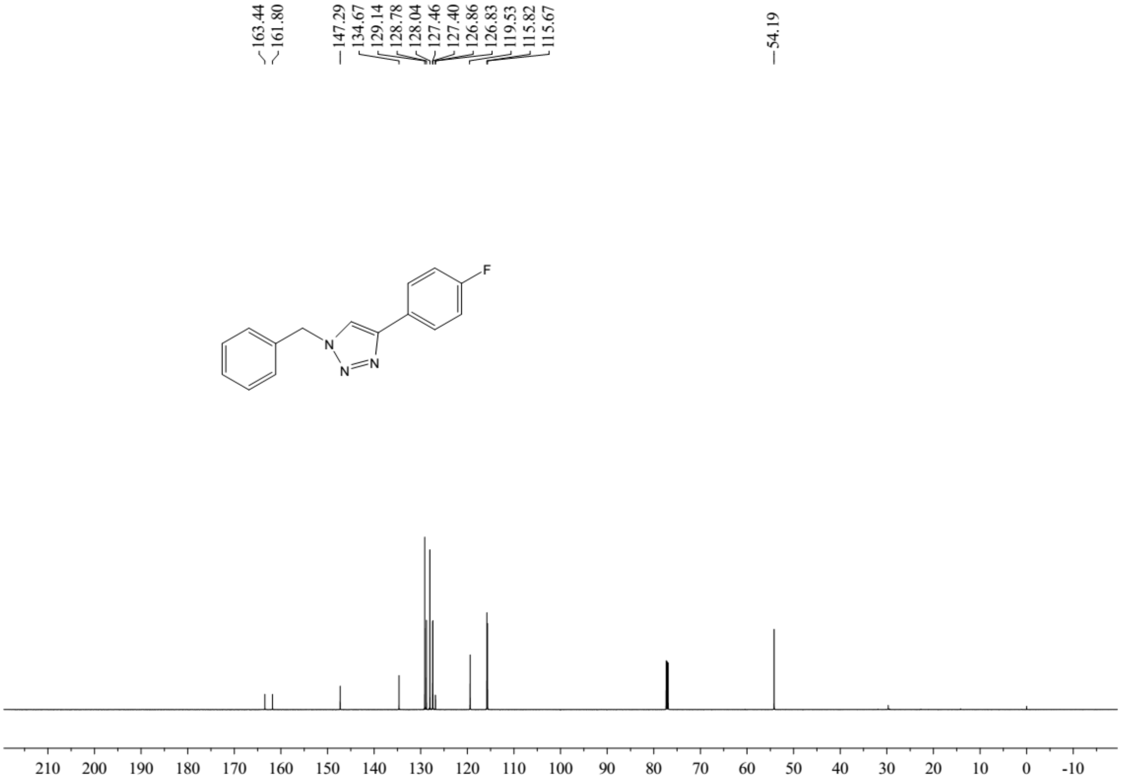
163.44
161.80
147.29
134.67
129.14
128.78
128.04
127.46
127.40
126.86
126.83
119.53
115.82
115.67
54.19
F
N
N
N
210 200 190 180 170 160 150 140 130 120 110 100 90 80 70 60 50 40 30 20 10 0 -10

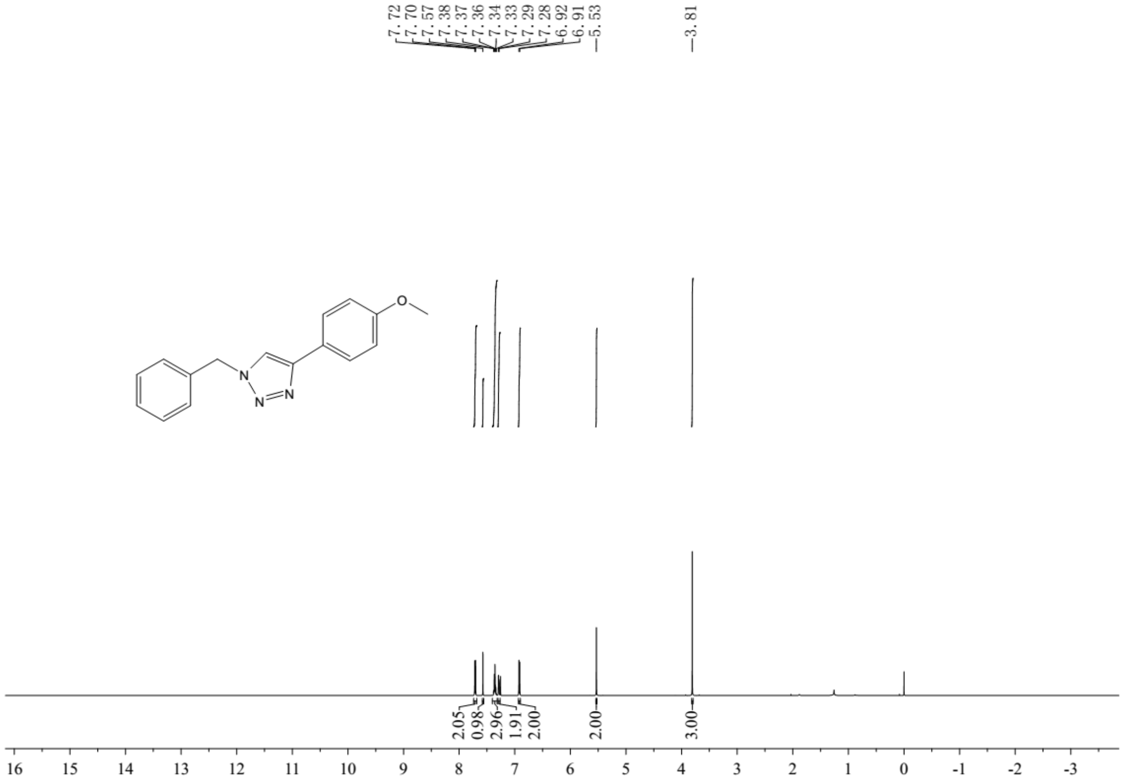
7.72
7.70
7.57
7.38
7.37
7.36
7.34
7.33
7.29
7.28
6.92
6.91
5.53
3.81
N
N
N
O
2.05
0.98
2.96
1.91
2.00
2.00
3.00
16 15 14 13 12 11 10 9 8 7 6 5 4 3 2 1 0 -1 -2 -3

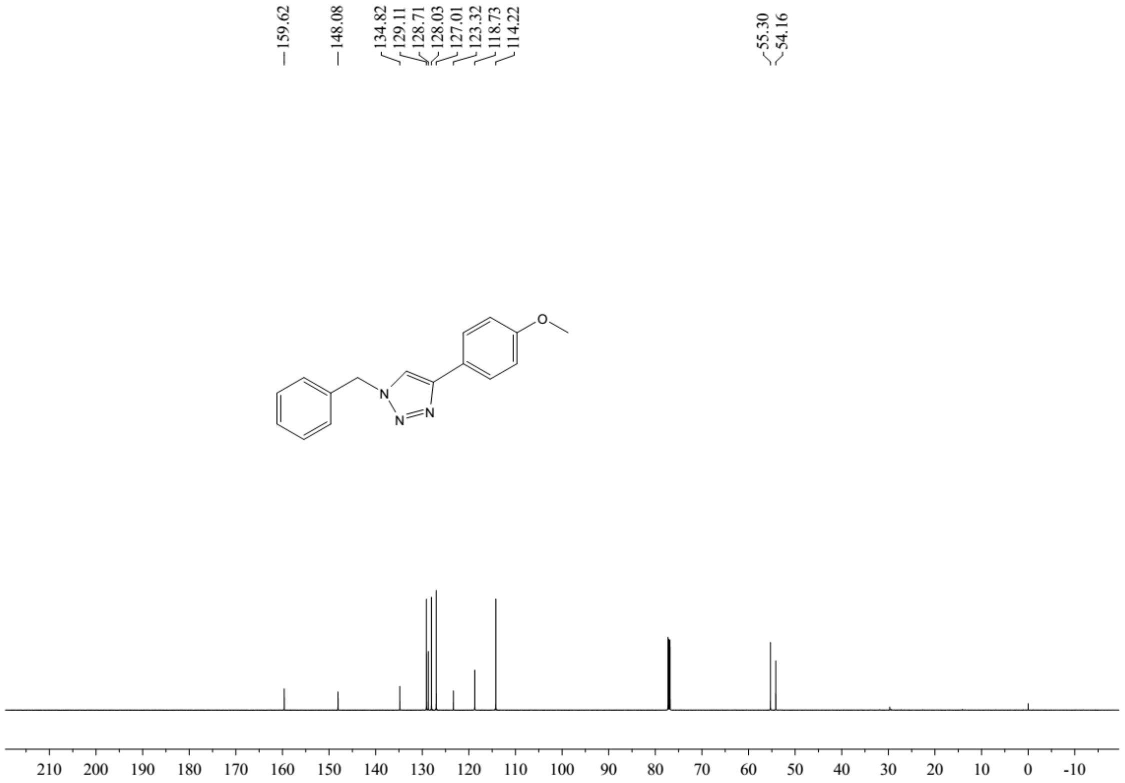
159.62
148.08
134.82
129.11
128.71
128.03
127.01
123.32
118.73
114.22
55.30
54.16
N
N
N
O
210 200 190 180 170 160 150 140 130 120 110 100 90 80 70 60 50 40 30 20 10 0 -10

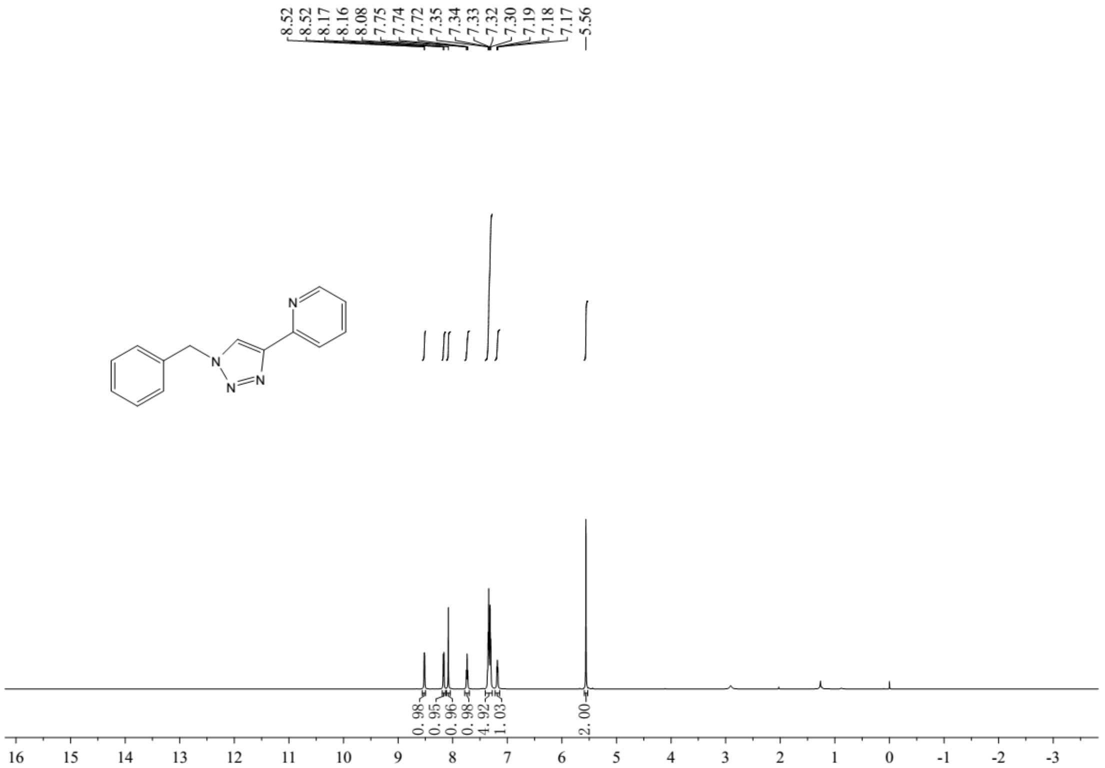
8.52
8.52
8.17
8.16
8.08
7.75
7.74
7.72
7.35
7.34
7.33
7.32
7.30
7.19
7.18
7.17
5.56
N
N
N
N
0.98
0.95
0.96
0.98
4.92
1.03
2.00
16 15 14 13 12 11 10 9 8 7 6 5 4 3 2 1 0 -1 -2 -3

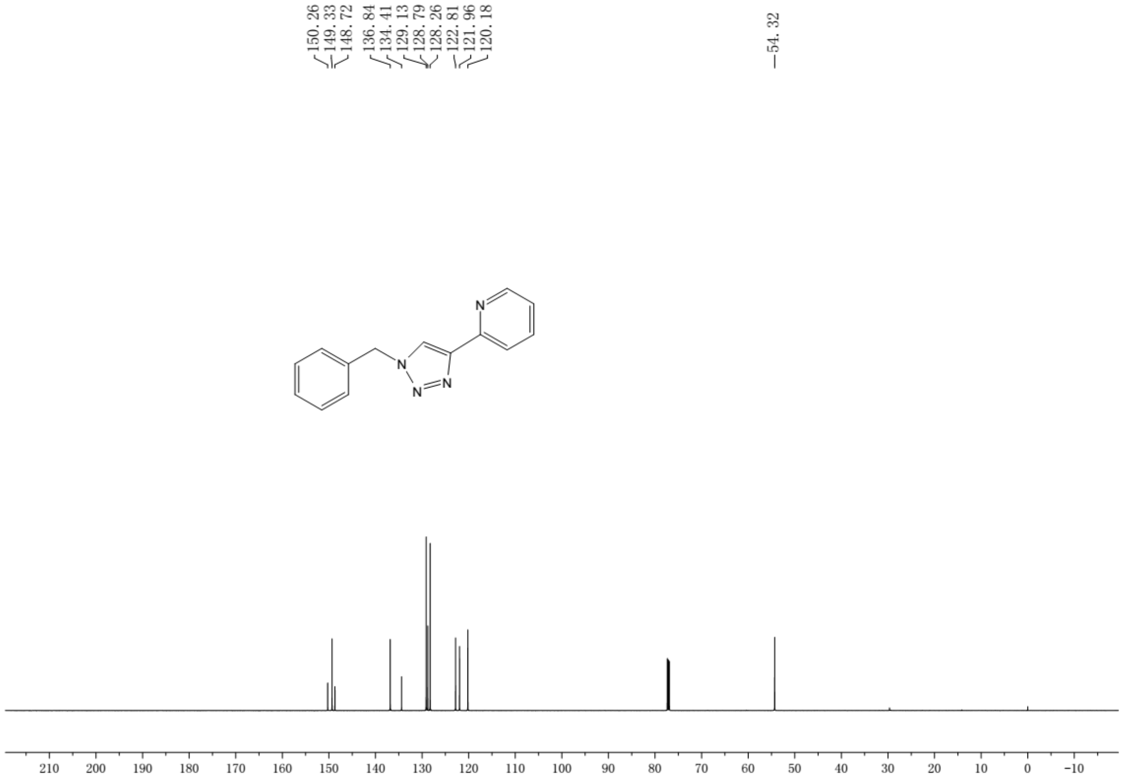
150.26
149.33
148.72
136.84
134.41
129.13
128.79
128.26
122.81
121.96
120.18
54.32
N
N
N
N
210 200 190 180 170 160 150 140 130 120 110 100 90 80 70 60 50 40 30 20 10 0 -10

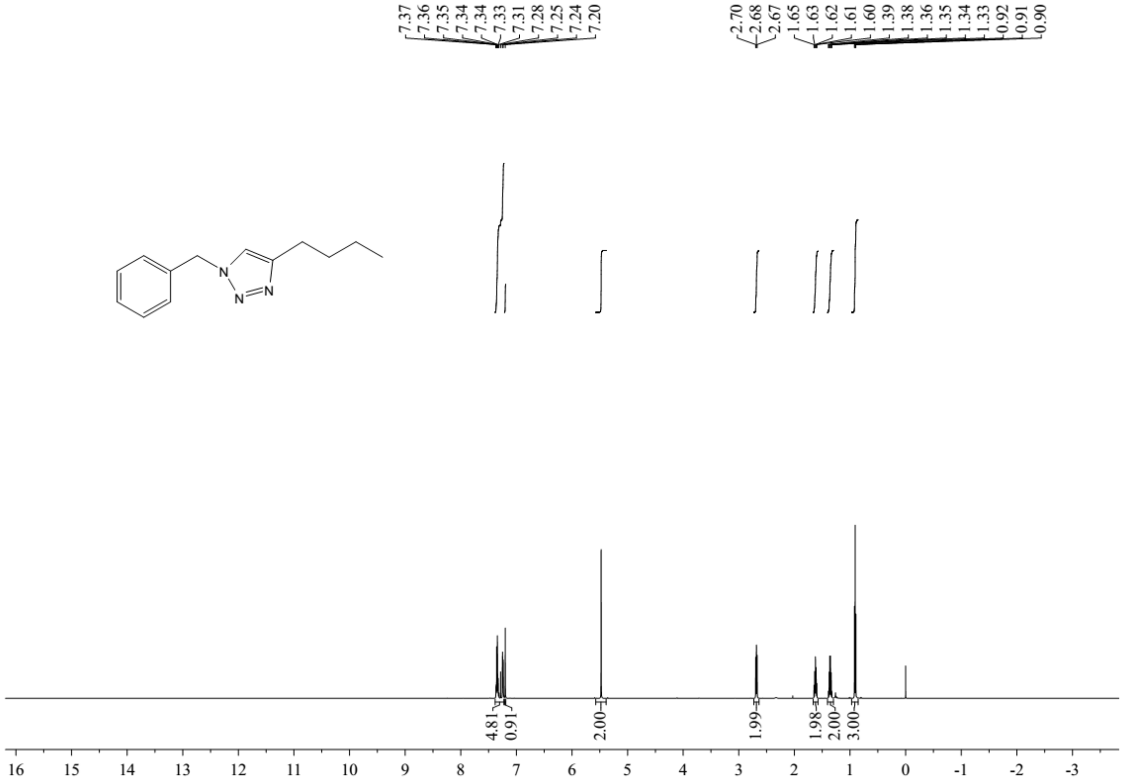
7.37 7.36 7.35 7.34 7.34 7.33 7.31 7.28 7.25 7.24 7.20
2.70 2.68 2.67 1.65 1.63 1.62 1.61 1.60 1.39 1.38 1.36 1.35 1.34 1.33 0.92 0.91 0.90
N
N
N
4.81 0.91 2.00 1.99 1.98 2.00 3.00
16 15 14 13 12 11 10 9 8 7 6 5 4 3 2 1 0 -1 -2 -3

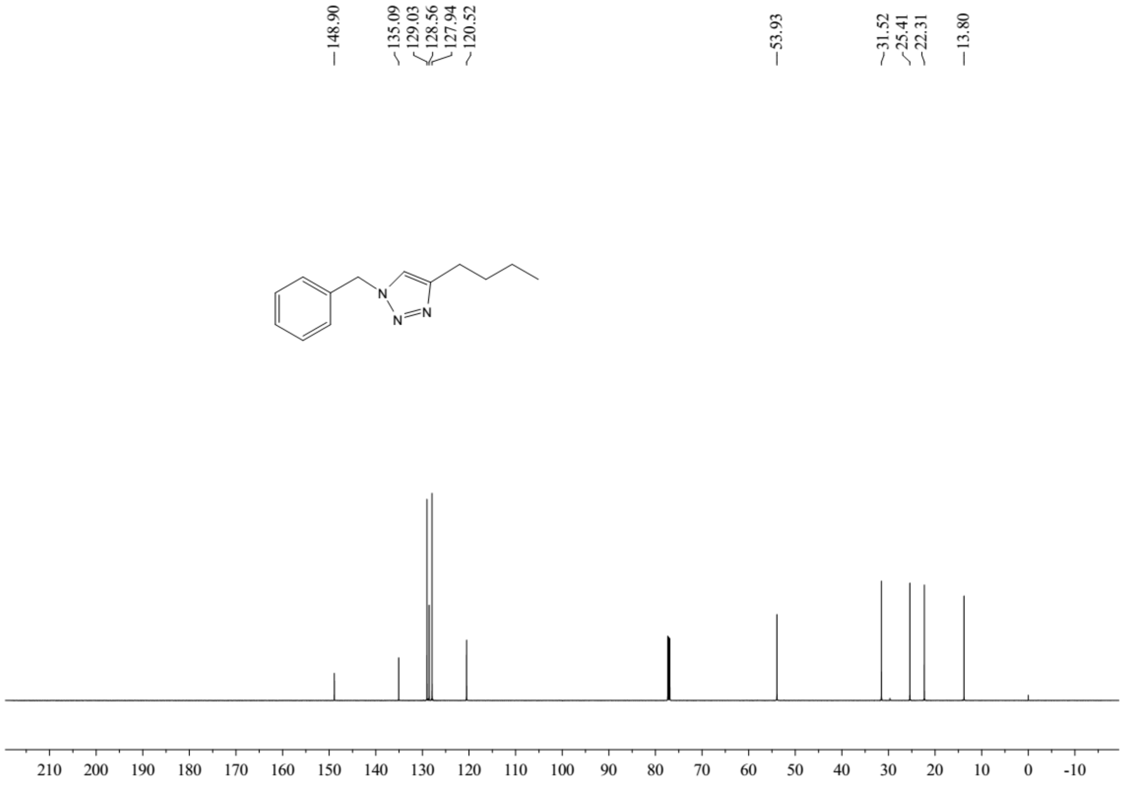
148.90
135.09 129.03 128.56 127.94 120.52
53.93
31.52 25.41 22.31
13.80
N
N
N
210 200 190 180 170 160 150 140 130 120 110 100 90 80 70 60 50 40 30 20 10 0 -10

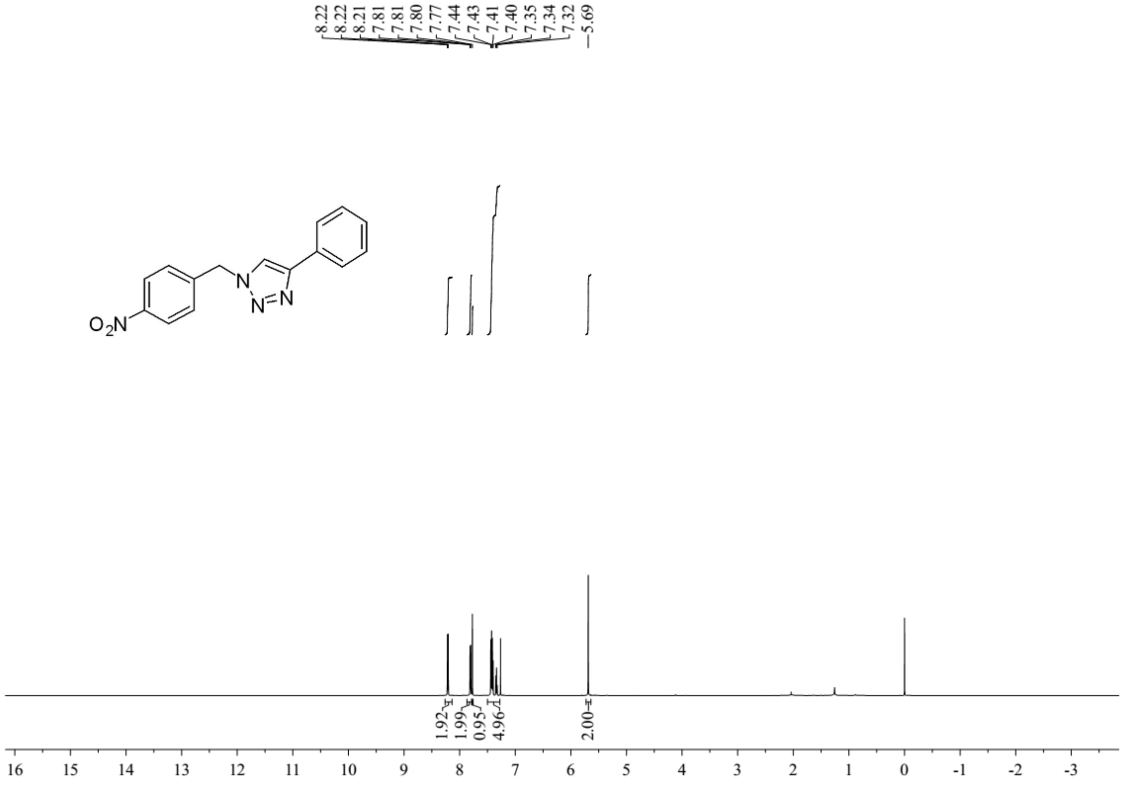

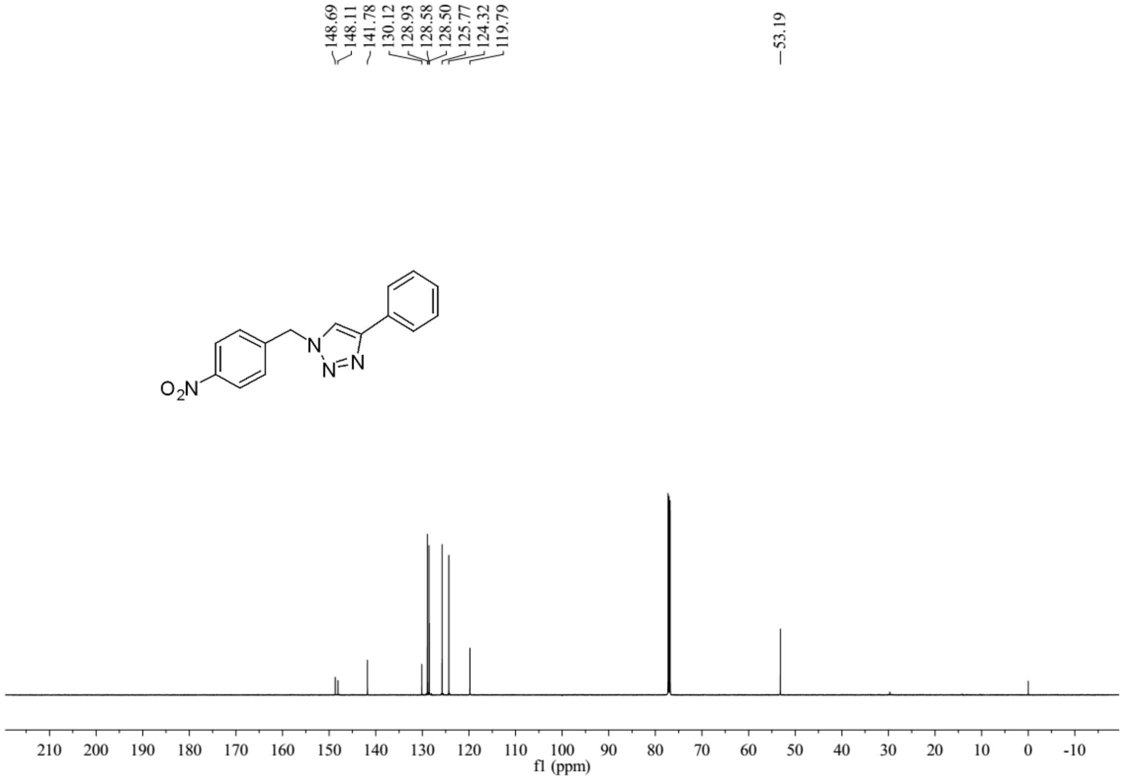

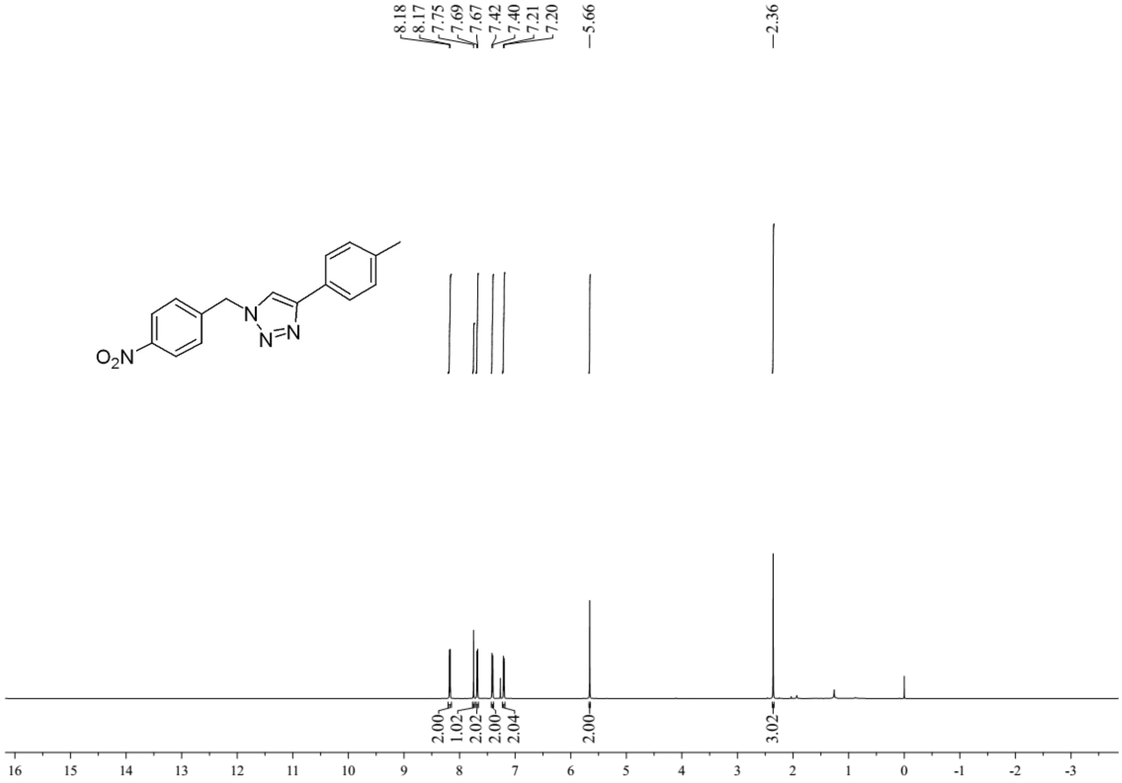
8.18
8.17
7.75
7.69
7.67
7.42
7.40
7.21
7.20
5.66
2.36
O_2N
N
N
N
2.00
1.02
2.02
2.00
2.04
2.00
3.02
16 15 14 13 12 11 10 9 8 7 6 5 4 3 2 1 0 -1 -2 -3

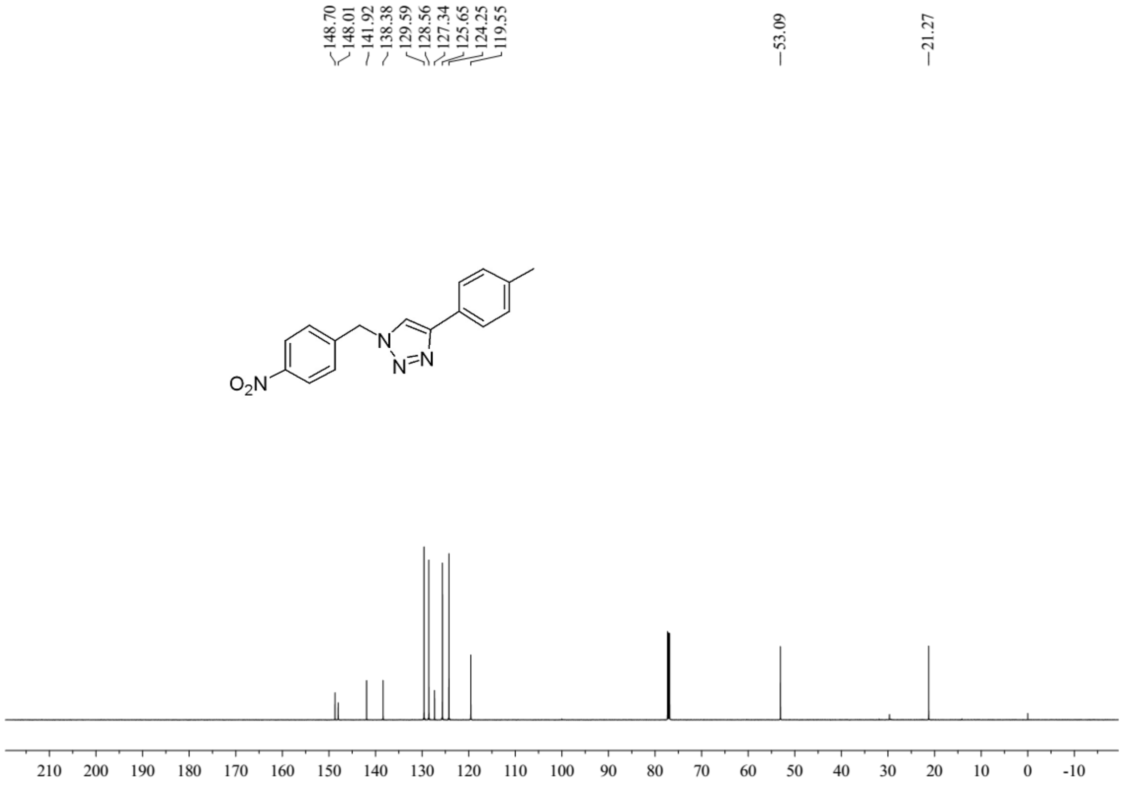
148.70
148.01
141.92
138.38
129.59
128.56
127.34
125.65
124.25
119.55
53.09
21.27
O_2N
N
N
N
210 200 190 180 170 160 150 140 130 120 110 100 90 80 70 60 50 40 30 20 10 0 -10

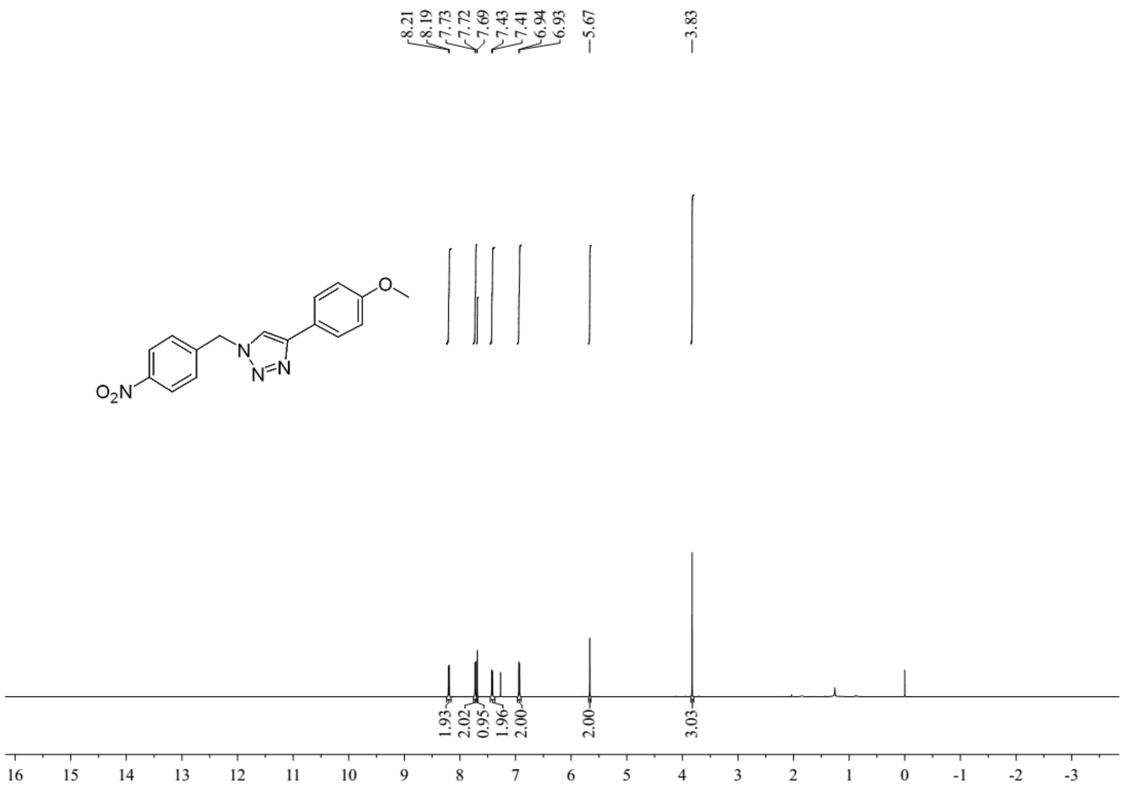
8.21
8.19
7.73
7.72
7.69
7.43
7.41
6.94
6.93
5.67
3.83
O2N
N
N=N
1.93
2.02
0.95
1.96
2.00
2.00
3.03
16 15 14 13 12 11 10 9 8 7 6 5 4 3 2 1 0 -1 -2 -3

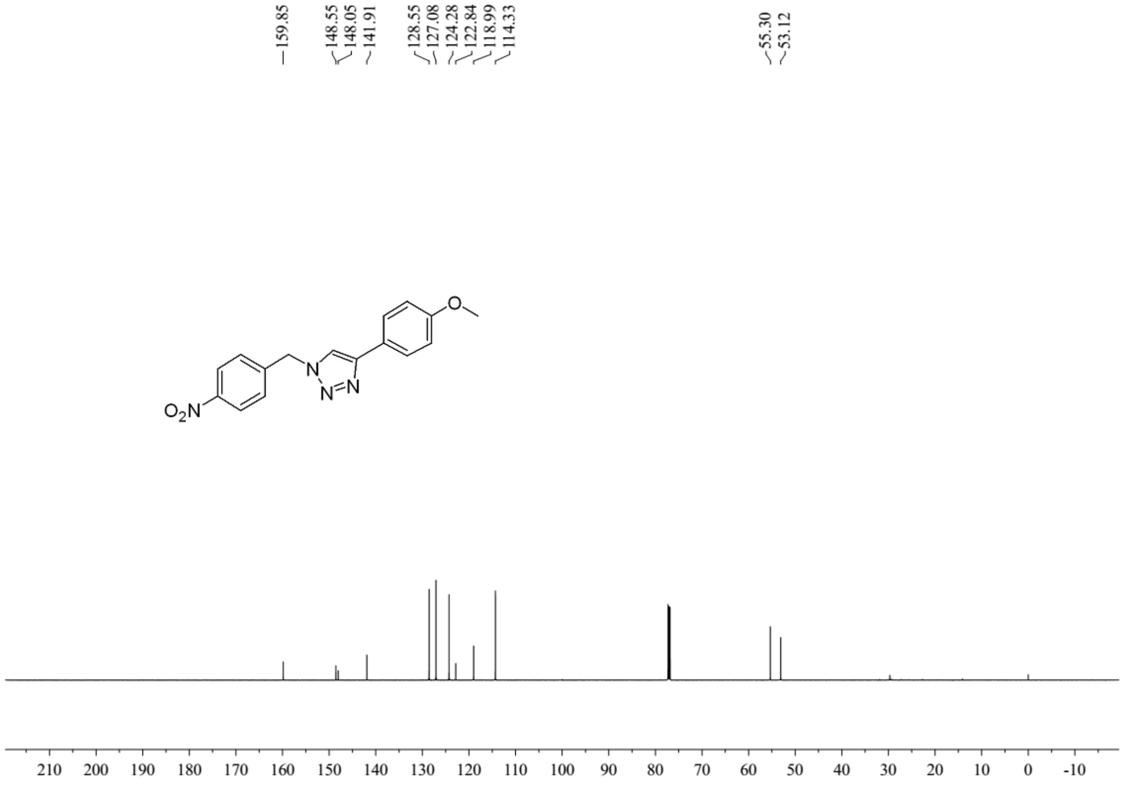
159.85
148.55
148.05
141.91
128.55
127.08
124.28
122.84
118.99
114.33
55.30
53.12
O2N
N
N=N
210 200 190 180 170 160 150 140 130 120 110 100 90 80 70 60 50 40 30 20 10 0 -10

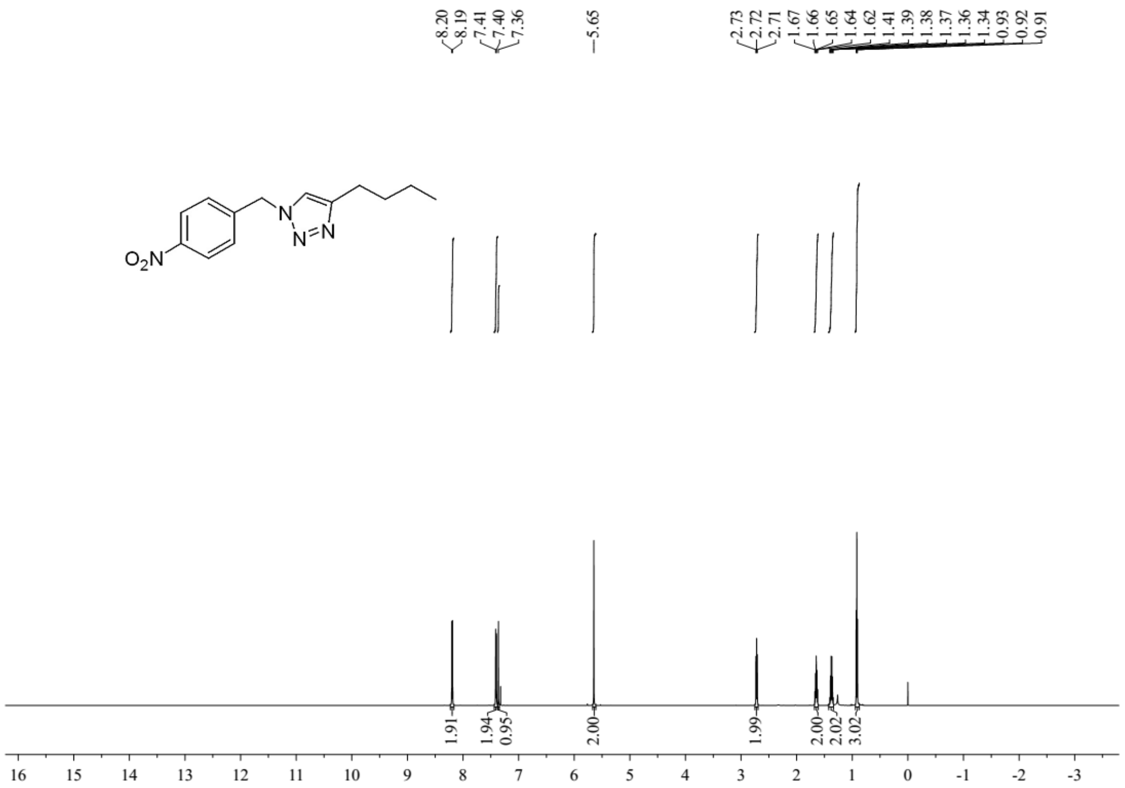
8.20
8.19
7.41
7.40
7.36
5.65
2.73
2.72
2.71
1.67
1.66
1.65
1.64
1.62
1.41
1.39
1.38
1.37
1.36
1.34
0.93
0.92
0.91
O_2N
N
N=N
1.91
1.94
0.95
2.00
1.99
2.00
2.02
3.02
16 15 14 13 12 11 10 9 8 7 6 5 4 3 2 1 0 -1 -2 -3

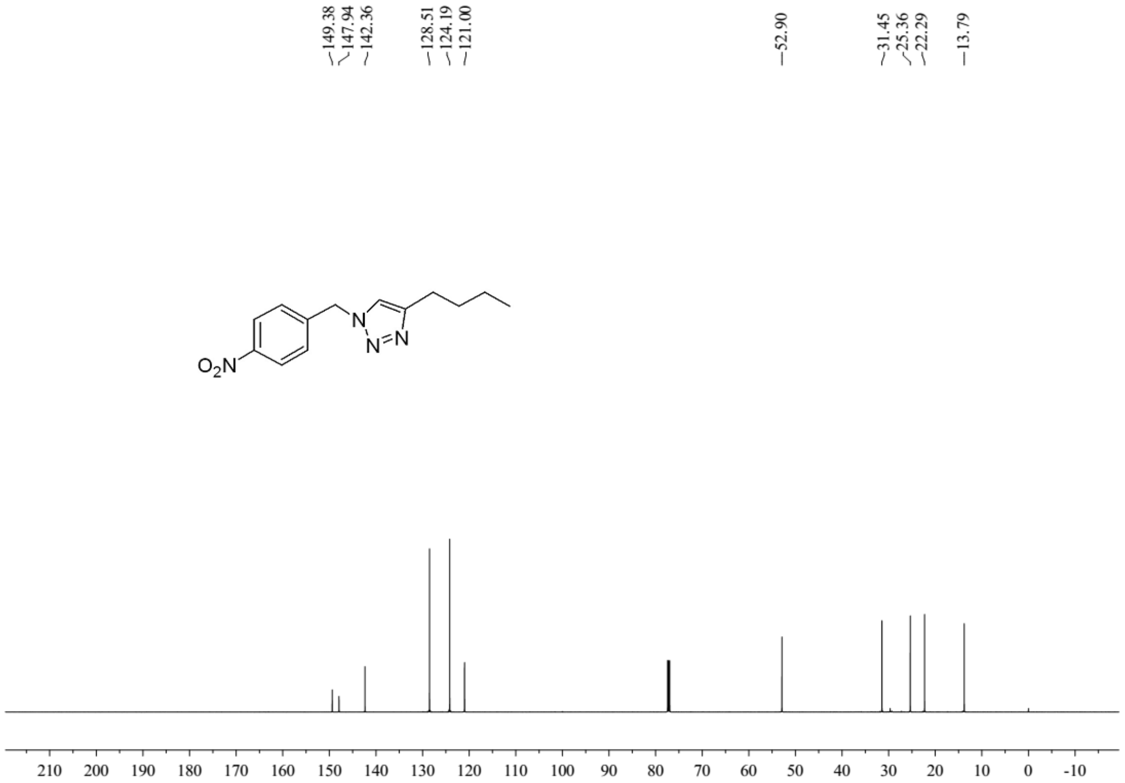
149.38
147.94
142.36
128.51
124.19
121.00
52.90
31.45
25.36
22.29
13.79
O_2N
N
N=N
210 200 190 180 170 160 150 140 130 120 110 100 90 80 70 60 50 40 30 20 10 0 -10

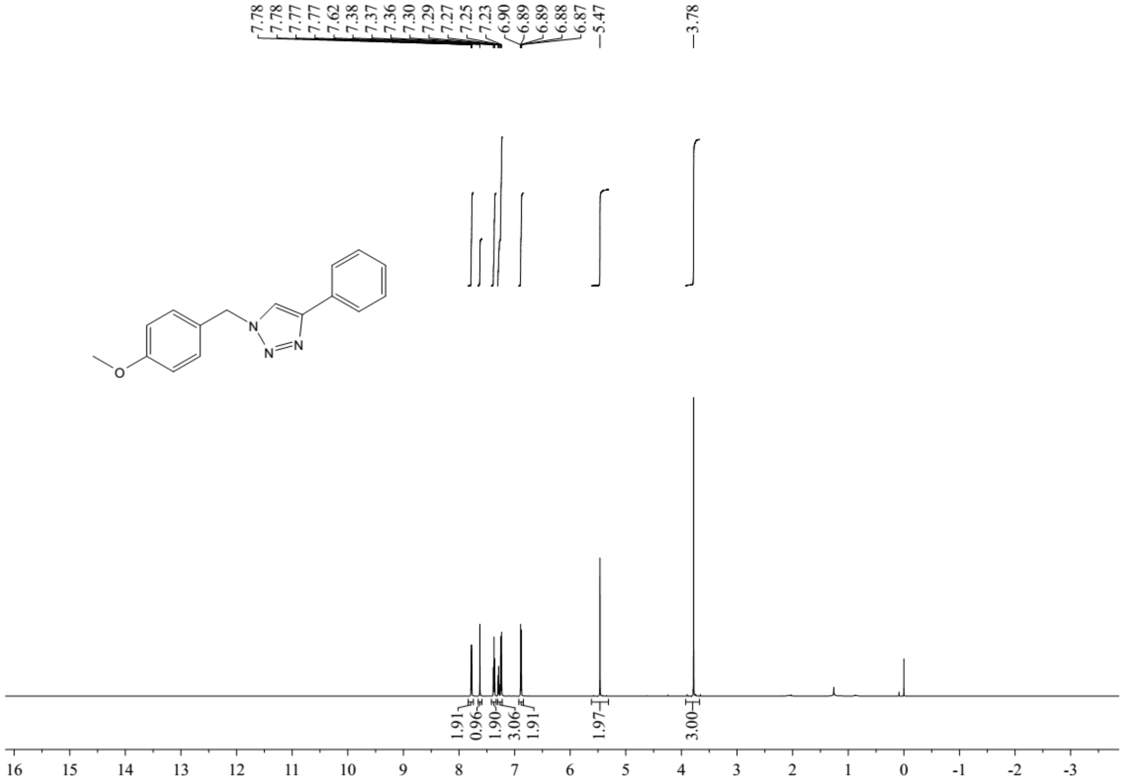
7.78
7.78
7.77
7.77
7.62
7.38
7.37
7.36
7.30
7.29
7.27
7.25
7.23
6.90
6.89
6.89
6.88
6.87
5.47
3.78
N
N
N
O
1.91
0.96
1.90
3.06
1.91
1.97
3.00
16 15 14 13 12 11 10 9 8 7 6 5 4 3 2 1 0 -1 -2 -3

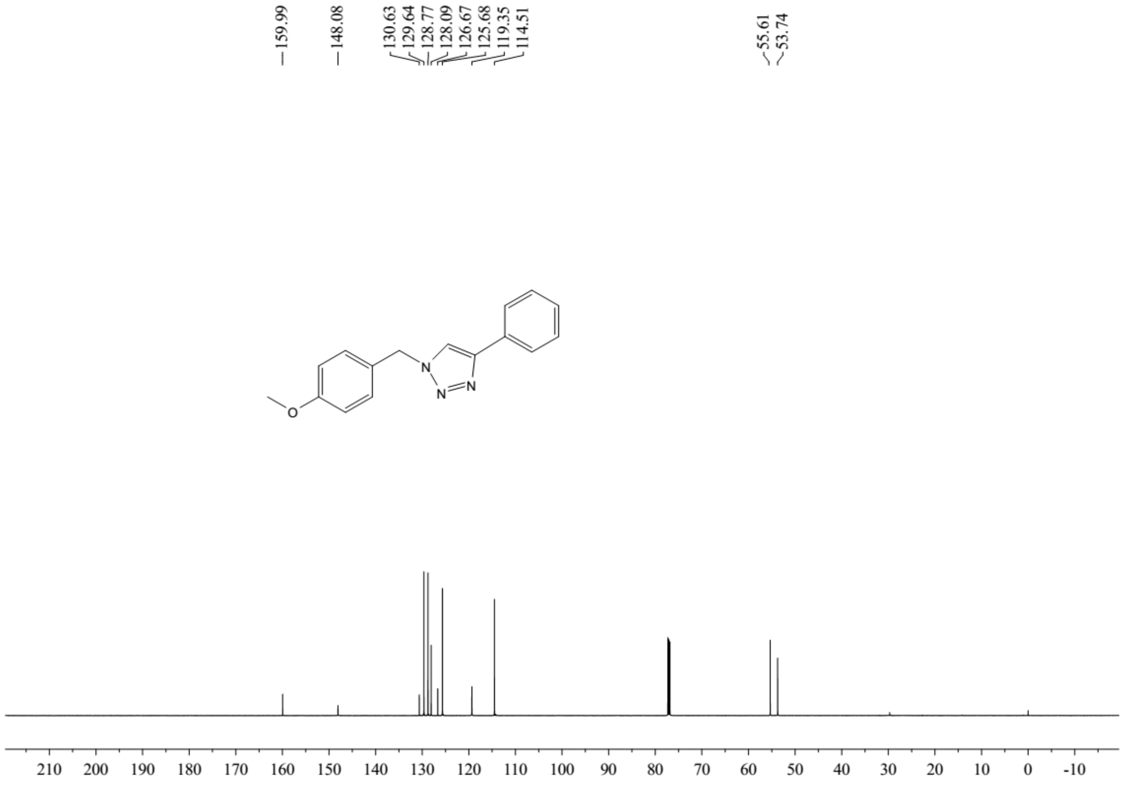
159.99
148.08
130.63
129.64
128.77
128.09
126.67
125.68
119.35
114.51
55.61
53.74
N
N
N
O
210 200 190 180 170 160 150 140 130 120 110 100 90 80 70 60 50 40 30 20 10 0 -10

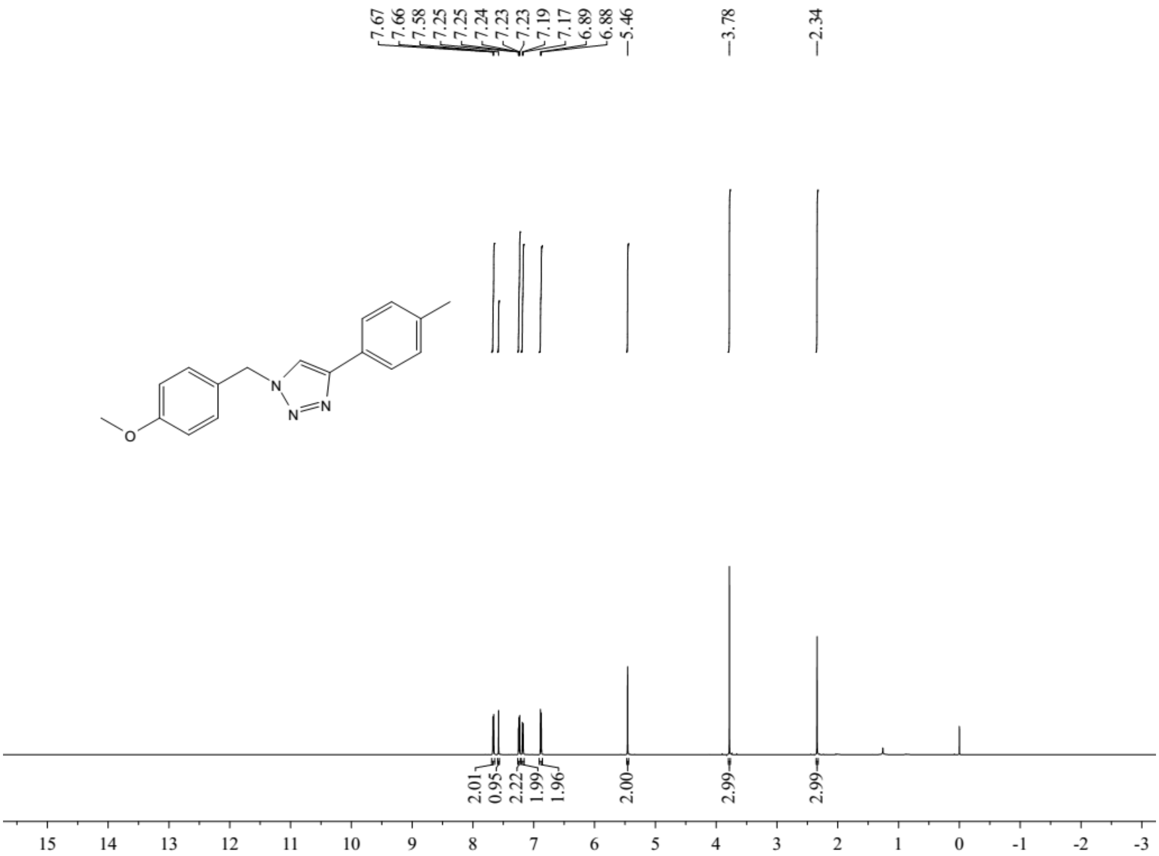
7.67
7.66
7.58
7.25
7.25
7.24
7.23
7.23
7.19
7.17
6.89
6.88
5.46
3.78
2.34
N
N
N
O
2.01
0.95
2.22
1.99
1.96
2.00
2.99
2.99
15 14 13 12 11 10 9 8 7 6 5 4 3 2 1 0 -1 -2 -3

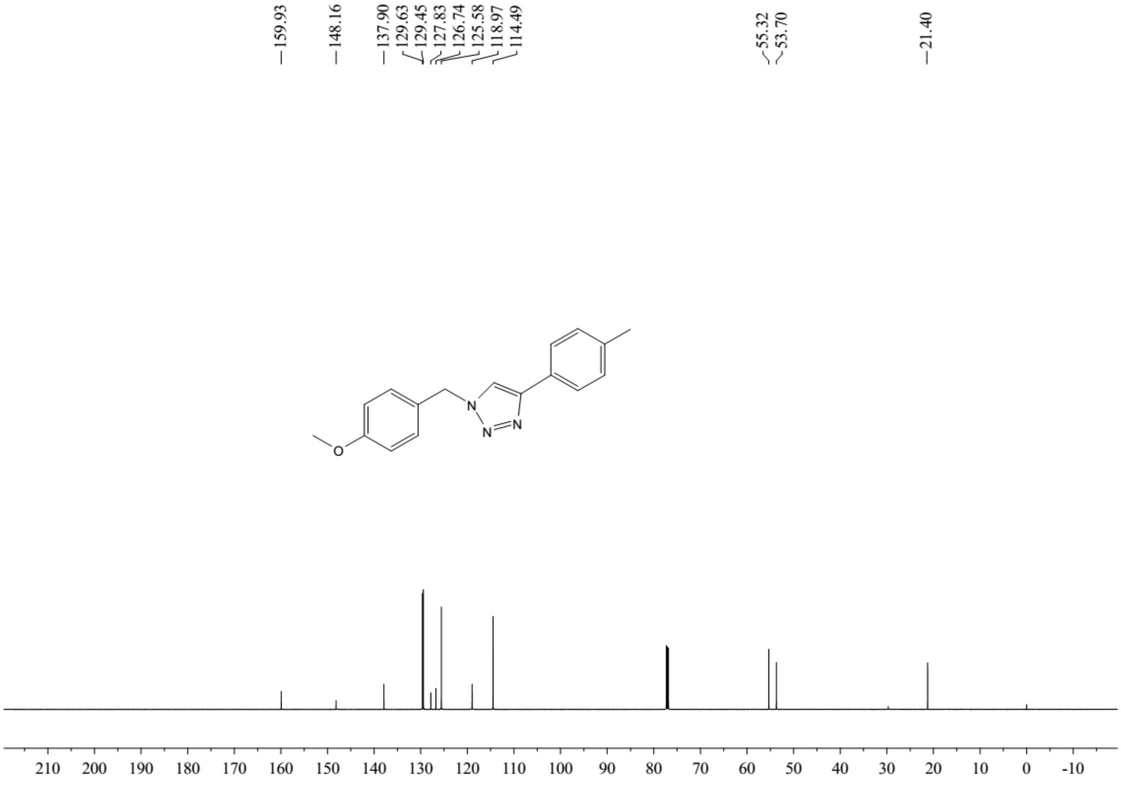
159.93
148.16
137.90
129.63
129.45
127.83
126.74
125.58
118.97
114.49
55.32
53.70
21.40
N
N
N
O
210 200 190 180 170 160 150 140 130 120 110 100 90 80 70 60 50 40 30 20 10 0 -10

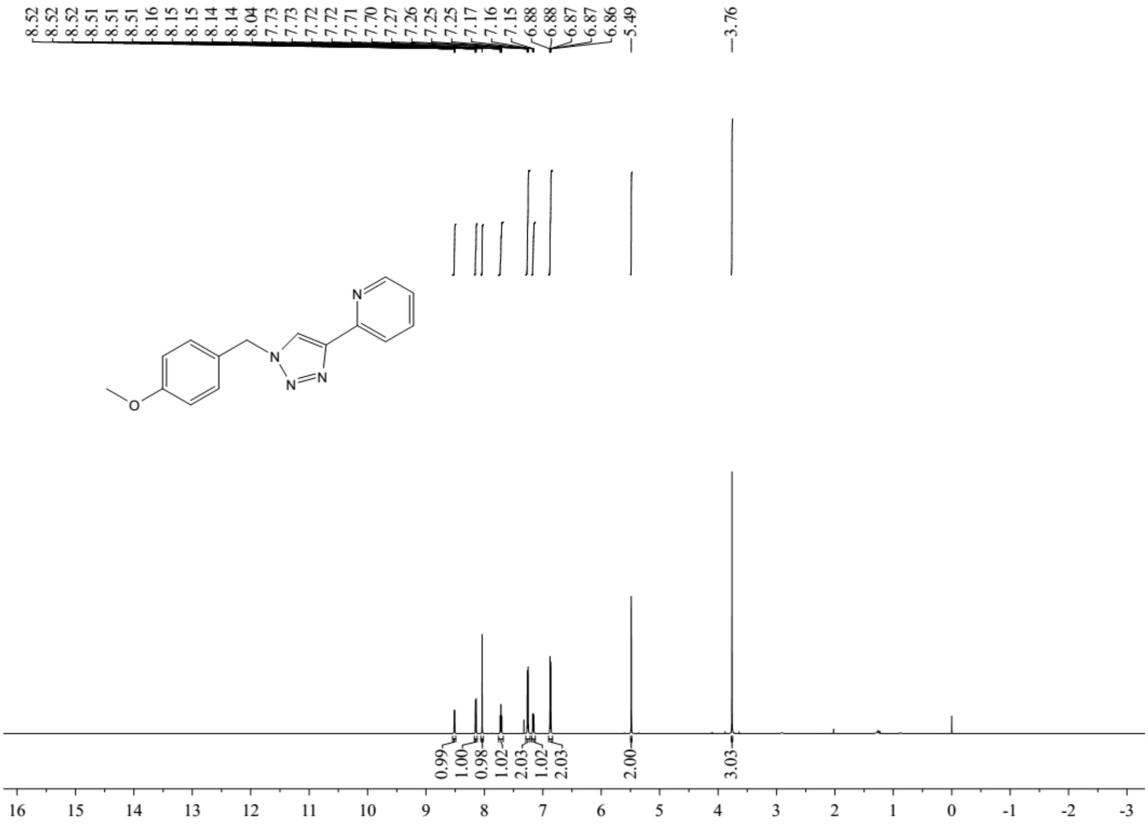
8.52 8.52 8.52 8.51 8.51 8.51 8.16 8.15 8.15 8.14 8.14 8.04 7.73 7.73 7.72 7.72 7.71 7.71 7.70 7.27 7.26 7.25 7.25 7.17 7.16 7.15 6.88 6.88 6.87 6.87 6.86 5.49
3.76
N
N
N
N
O
0.99 1.00 0.98 1.02 2.03 1.02 2.03
2.00
3.03
16 15 14 13 12 11 10 9 8 7 6 5 4 3 2 1 0 -1 -2 -3

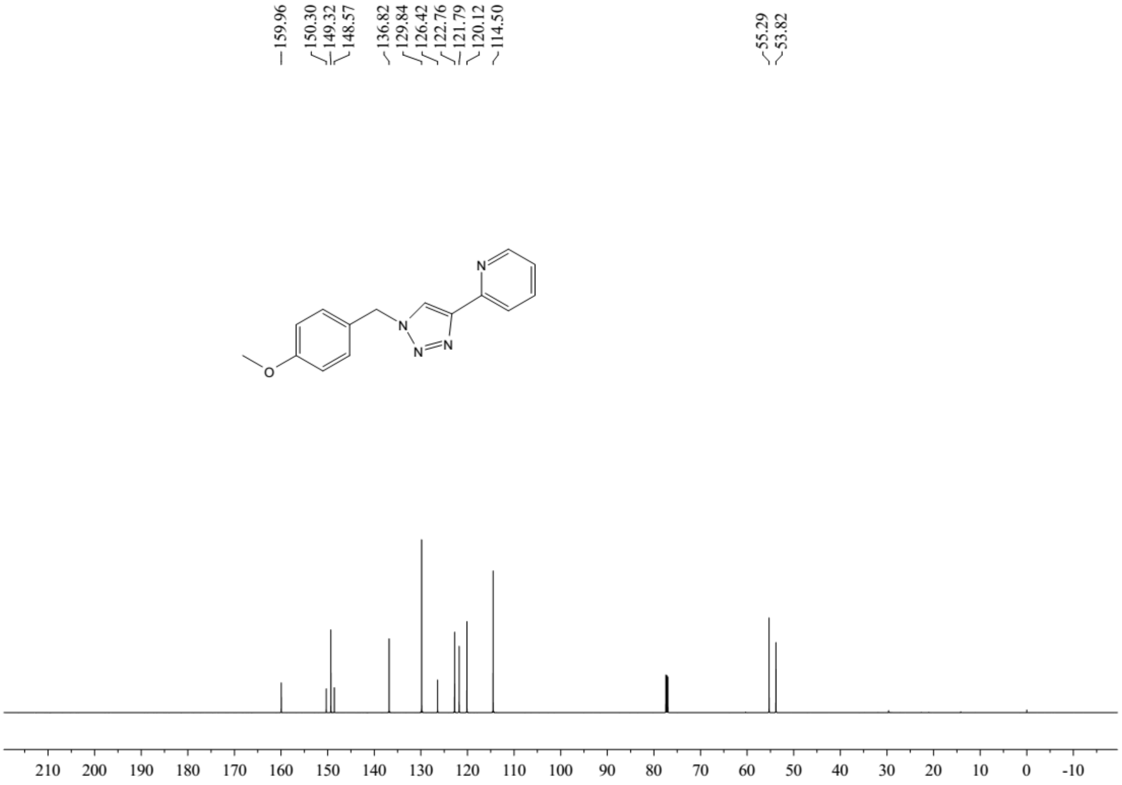
159.96
150.30 149.32 148.57
136.82 129.84 126.42 122.76 121.79 120.12 114.50
55.29 53.82
N
N
N
N
O
210 200 190 180 170 160 150 140 130 120 110 100 90 80 70 60 50 40 30 20 10 0 -10

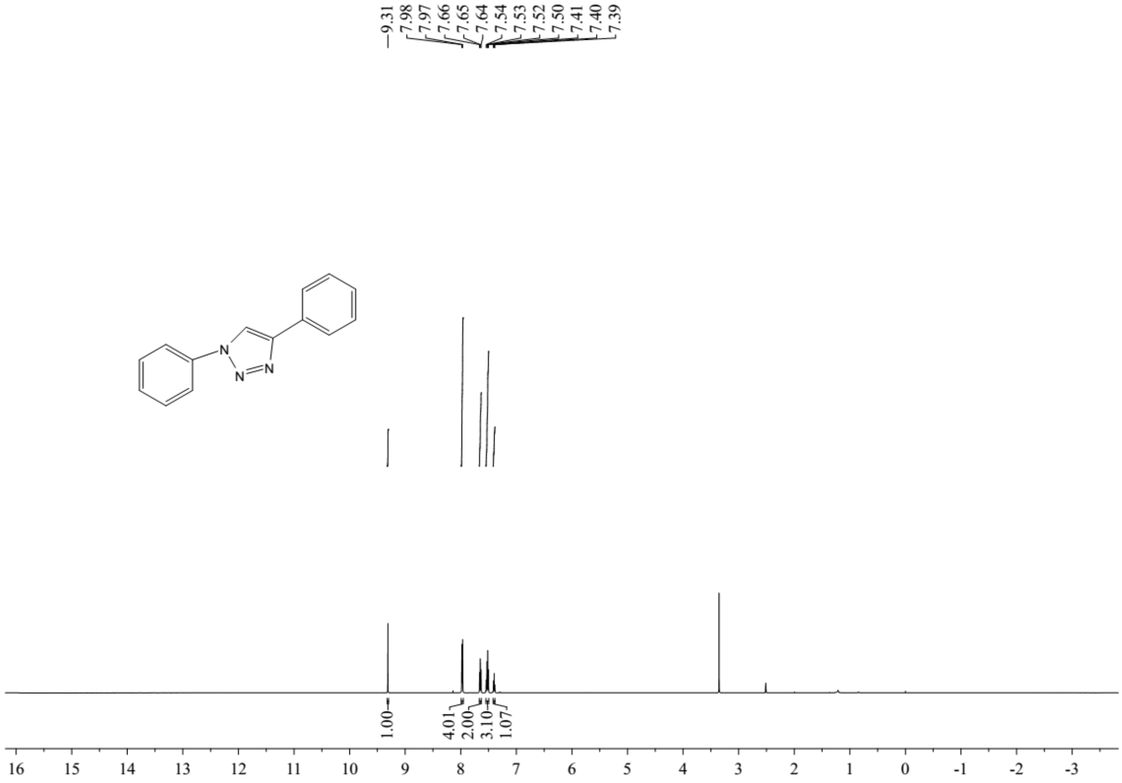
9.31
7.98
7.97
7.66
7.65
7.64
7.54
7.53
7.52
7.50
7.41
7.40
7.39
N
N
N
1.00
4.01
2.00
3.10
1.07
16 15 14 13 12 11 10 9 8 7 6 5 4 3 2 1 0 -1 -2 -3

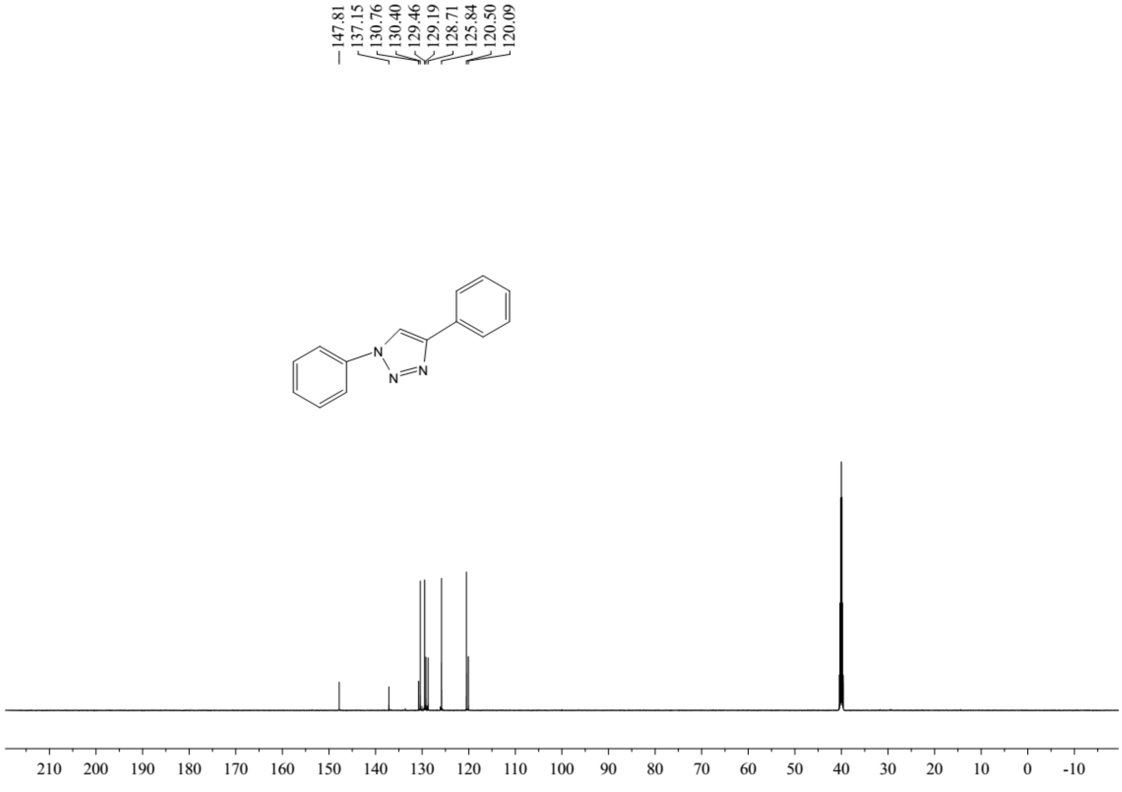
147.81
137.15
130.76
130.40
129.46
129.19
128.71
125.84
120.50
120.09
N
N
N
210 200 190 180 170 160 150 140 130 120 110 100 90 80 70 60 50 40 30 20 10 0 -10

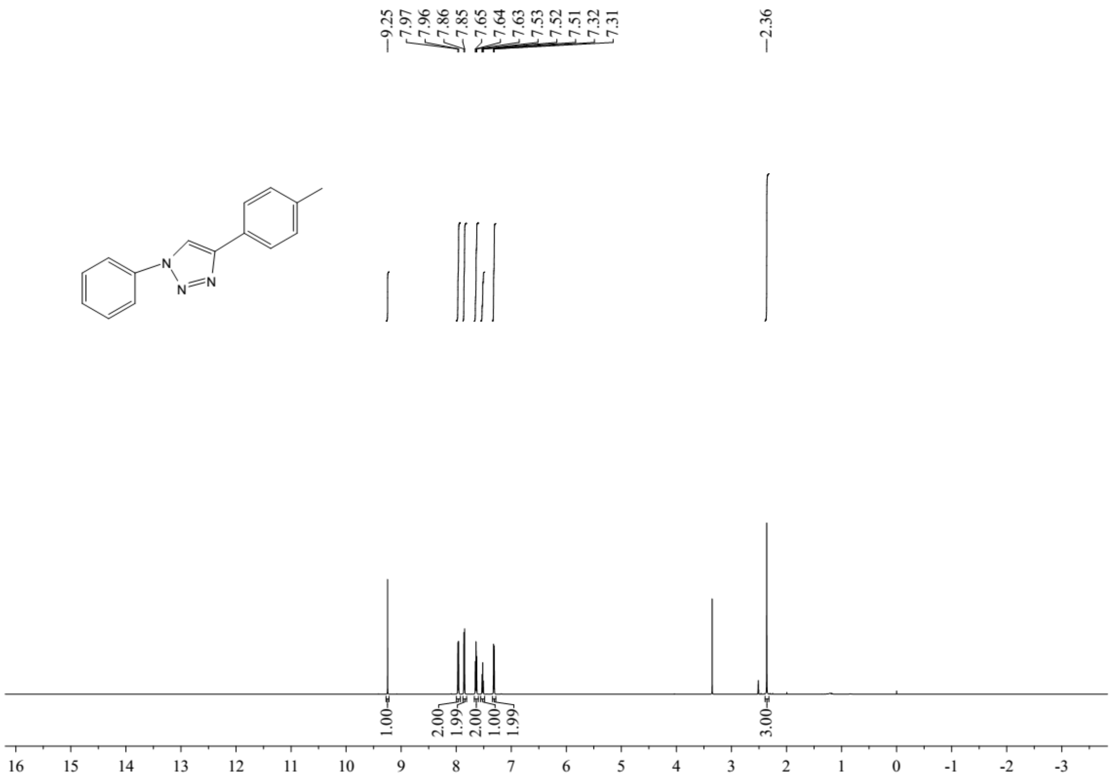
9.25
7.97
7.96
7.86
7.85
7.65
7.64
7.63
7.53
7.52
7.51
7.32
7.31
2.36
N
N
N
1.00
2.00
1.99
2.00
1.00
1.99
3.00

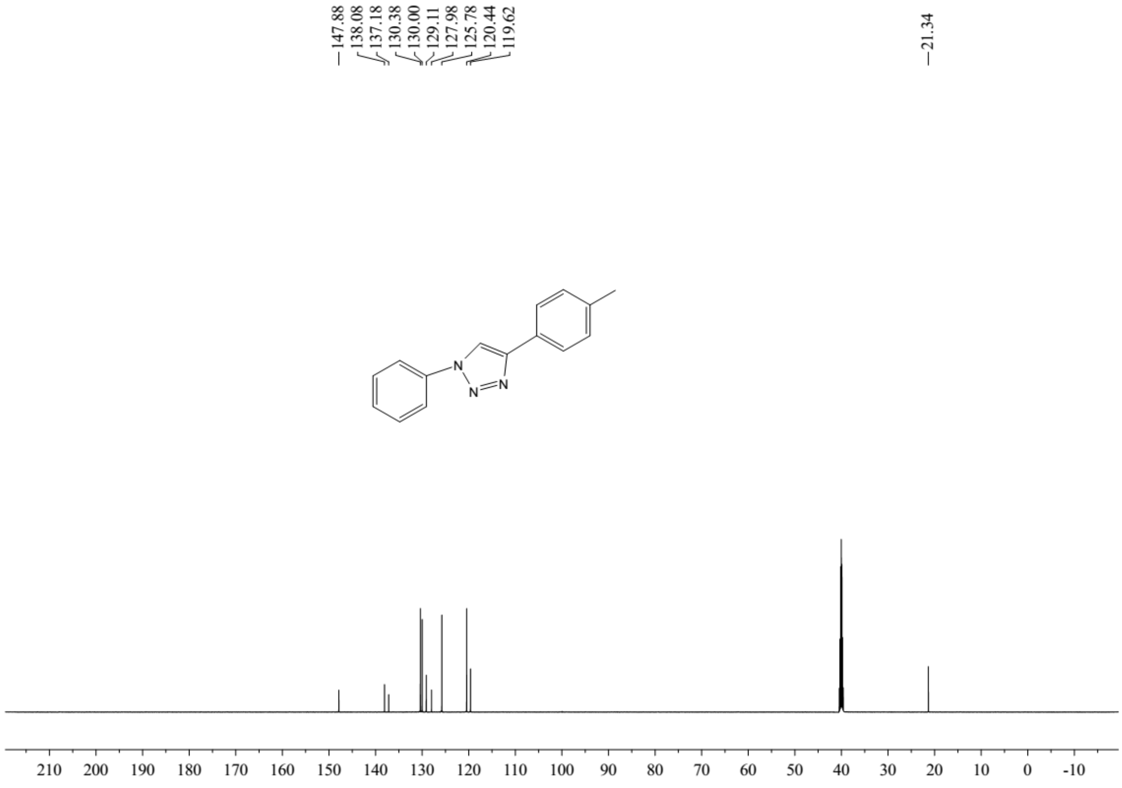
147.88
138.08
137.18
130.38
130.00
129.11
127.98
125.78
120.44
119.62
21.34
N
N
N

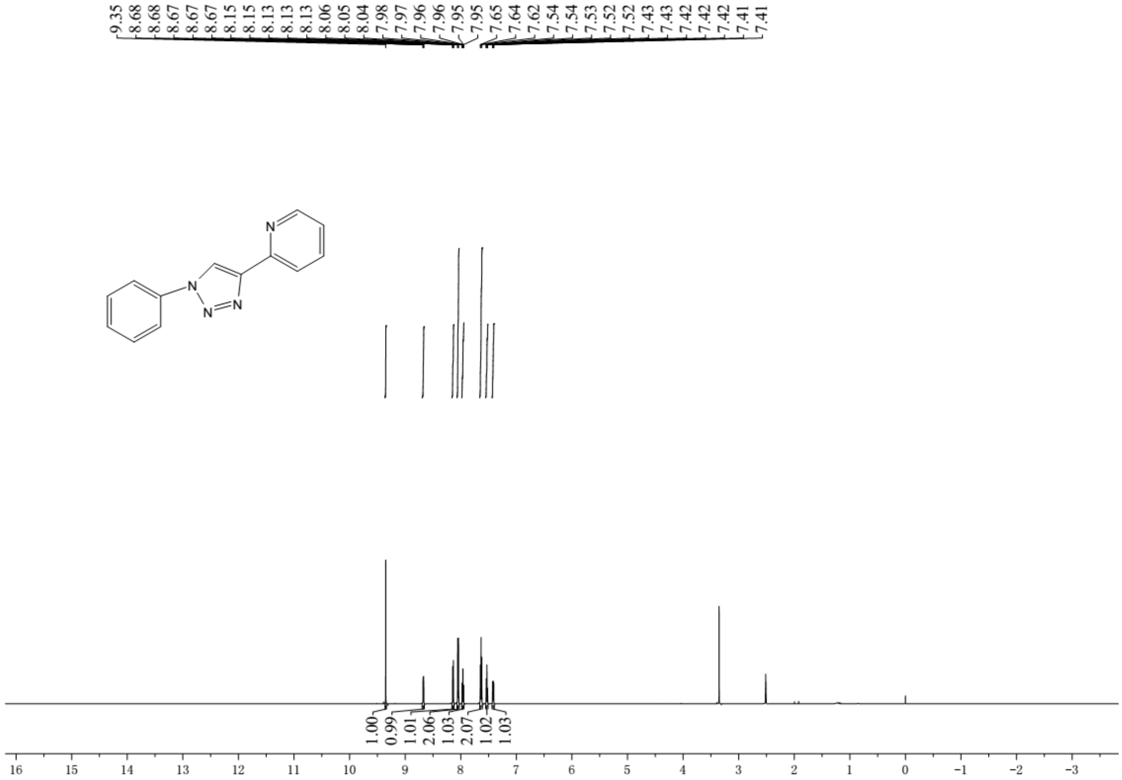
9.35 8.68 8.68 8.67 8.67 8.67 8.15 8.15 8.13 8.13 8.13 8.06 8.05 8.04 7.98 7.97 7.96 7.96 7.95 7.95 7.65 7.64 7.62 7.54 7.54 7.53 7.52 7.52 7.43 7.43 7.42 7.42 7.42 7.41 7.41
N
N
N=N
1.00 0.99 1.01 2.06 1.03 2.07 1.02 1.03
16 15 14 13 12 11 10 9 8 7 6 5 4 3 2 1 0 −1 −2 −3

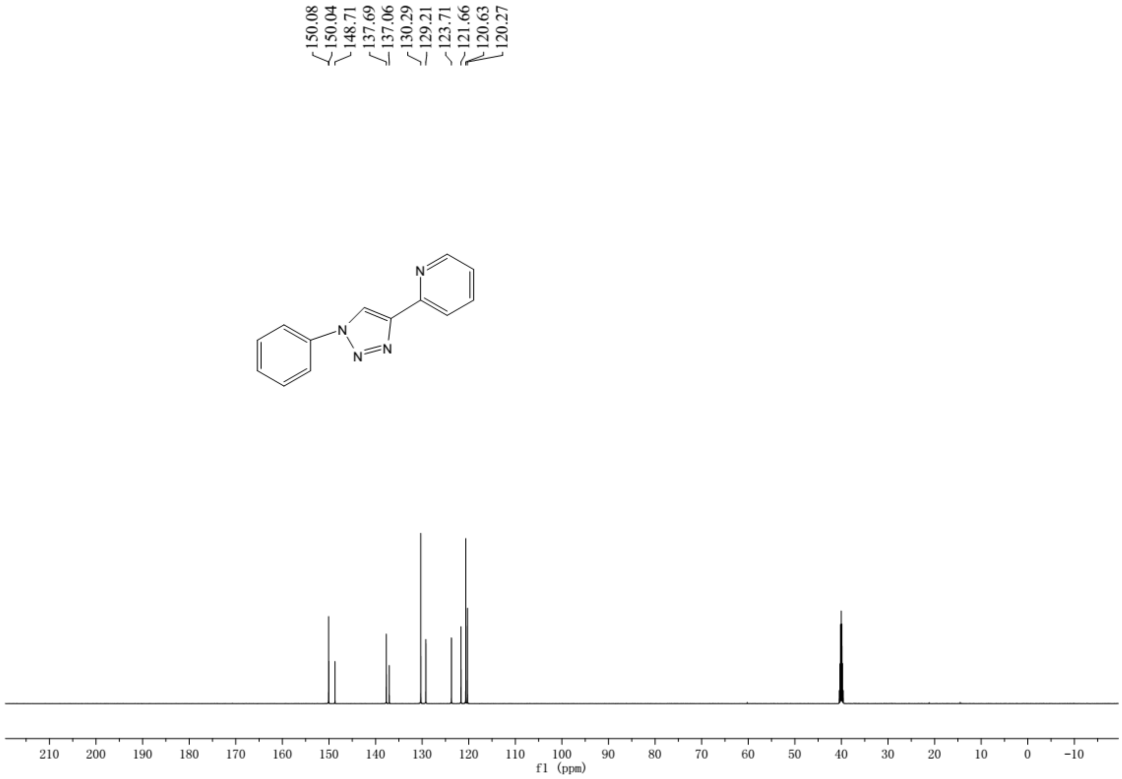
150.08 150.04 148.71 137.69 137.06 130.29 129.21 123.71 121.66 120.63 120.27
N
N
N=N
210 200 190 180 170 160 150 140 130 120 110 100 90 80 70 60 50 40 30 20 10 0 −10
f1 (ppm)
